# Coaching as a Developmental Intervention in Organisations: A Systematic Review of Its Effectiveness and the Mechanisms Underlying It

**DOI:** 10.1371/journal.pone.0159137

**Published:** 2016-07-14

**Authors:** Simmy Grover, Adrian Furnham

**Affiliations:** 1 Research Department of Clinical, Educational and Health Psychology, University College London, 26 Bedford Way, London, WC1H0AP, United Kingdom; 2 Norwegian Business School (BI), Nydalveien, Olso, Norway; Defence Science and Technology Group, AUSTRALIA

## Abstract

**Purpose:**

The primary aim of this paper is to conduct a thorough and systematic review of the empirical and practitioner research on executive, leadership and business coaching to assess the current empirical evidence for the effectiveness of coaching and the mechanisms underlying it.

**Background:**

Organisations are increasingly using business coaching as an intervention to improve the productivity and performance of their senior personnel. A consequence of this increased application is the demand for empirical data to understand the process by which it operates and its demonstrable efficacy in achieving pre-set goals.

**Method:**

This paper is a systematic review of the academic and practitioner literature pertaining to the effectiveness of business and executive coaching as a developmental intervention for organisations. It focuses on published articles, conference papers and theses that cover business, leadership or executive coaching within organisations over the last 10 years.

**Conclusions:**

The main findings show that coaching is an effective tool that benefits organisations and a number of underlying facets contribute to this effectiveness. However, there is deficiency and scope for further investigation in key aspects of the academic research and we identify several areas that need further research and practitioner attention. ​

## Introduction

A recent global survey of coaches [1] estimated that the coaching industry is worth $2bn annually. This includes all forms of coaching: business as well as life and health. The survey also found that coaches are seeing increases in the number of hours they are working, the numbers of clients they have and the fees that they are charging. Furthermore, just over half of the coaches globally, about 28,000 are believed to work in the field of business, executive or leadership coaching. Clearly coaching is an established and significantly sized industry yet there remains still relatively little objective empirical evidence of its efficacy.

The use of coaching within organisational settings, whether it is classified as business, leadership or executive coaching, has grown substantially to become an established practice in a number of organisations and it continues to flourish [2]. It is used in organisational settings to improve employee, team and organisational performance in a number of ways, including but not limited to: helping shorten the learning curve in a new organization, country or role, succession planning and career planning, to improve job satisfaction, flexibility, interpersonal relationships, and leadership and management skills [3]. Furthermore, coaching can be implemented under a number of guises: external or internal coaching, team or peer coaching, telephone or e-coaching, manager as a coach or by creating a coaching culture.

Although coaching has become an established and popular intervention within organisations, there is limited evaluation of coaching programs by organisations [4] and little consensus among academics as to the best mechanism for evaluation [5]. As a result, the perceptions of the effectiveness of coaching differ widely: some believe there is “evidence of absence” in the sense that studies have shown it to be not very effective, while others argue there is in fact an “absence of evidence” in the sense that, as yet, few good studies have been done.

There is a very long history in clinical psychology and psychiatry of attempts to evaluate the efficacy of specific interventions [6]. It is recognized to be difficult and expensive research but crucially important not only to check the claims of practitioners but also to understand the processes and mechanisms underlying the therapies. Whilst there are many similarities (and differences) between counselling, coaching and psychotherapy there remains very few good empirical studies that examine “what works for whom” in the coaching world.

The main aim of this research is to create a foundation for those organisations and individuals interested in understanding the effectiveness of coaching interventions within a business setting. This review will systematically review the last 10 years of academic and practitioner research pertaining to the effectiveness of business, executive and leadership coaching in an organisational setting. With a goal to not only investigate whether coaching is effective as an intervention but also to understand the mechanisms that have been explored to potentially explain that effectiveness. Researchers have strongly advocated the use of both systematic reviews and meta-analyses to determine the overall efficacy of interventions as well as examining very specific types of intervention within a particular sphere. This paper reports a review of the rigorous papers in the area.

### Coaching: Historical Perspectives

A search of the literature pertaining to coaching yields thousands of articles from a number of domains, such as sports [7], health [8], education [9] and business [10]. Specifically in the organisational space, the term coaching first appeared in 1937 [11], however this paper was rather unique and only a few other papers were published over the next 50 years [10]. There was a spur in coaching research in the late-1980s and 1990s, and a comprehensive review of the executive coaching literature in 2001 [12] found seven empirical studies of executive coaching. Since this review in 2001 the growth of empirical studies examining executive and leadership coaching has grown considerably [10].

Coaching as an intervention, potentially due to its diverse and widespread application as well as the heterogeneity of its practitioners, has evolved as an amalgamation of a number of helping, development and training techniques. Early reviews in the space focused on establishing a definition of coaching and boundaries that separated it from counselling and consulting. Unsurprisingly, a multitude of definitions of coaching exist from practitioners, academics and associations with various attributes and specificity (see Maltbia, Marsick & Ghosh [13] for a list of definitions). However, a number of underlying themes emerge from these definitions, such as a one-on-one systematic relationship, non-clinical population, learning, behavioural change, self-awareness and improved performance [2, 10].

Within an organizational setting, a number of terms are used to describe coaching, such as “business coaching”, “executive coaching” and “leadership coaching”. Business coaching can be considered as an overarching term that refers to any individual within an organization receiving coaching to improve performance. Executive coaching is more specific in terms of the coachee involved: usually an executive or an individual from senior management. On the other hand, leadership coaching differs in terms of the goal or outcome of the coaching. It is a specific form of coaching that is centred around enabling the client/coachee to become a more effective leader [14].

In their 2001 review, Kampa-Kokesch and Anderson [12] found that four of the seven empirical studies, both qualitative and quantitative studies, had investigated some aspect related to the effectiveness of coaching. They concluded that these studies provided initial although limited evidence that coaching was an effective intervention for improving performance, facilitating behavioural change and was positively viewed by coachees. A follow up review of the executive coaching literature by Joo [2] in 2005 did not have many more additional studies to deal with. However, two more quantitative studies [15, 16] had been carried out and pointed towards the effectiveness of coaching in terms of improvements in other-ratings of performance.

A few years later a meta-analysis [17] was attempted to investigate the research evaluating the effectiveness of coaching. Although 22 studies were initially identified only six of them were included in the meta-analytic procedure because a number of the studies used poor methodological design, such as retrospective design, for example many of the authors just asked coachees, coaches or supervisors how effective they perceived coaching to be after the intervention. Additionally, some studies reported insufficient data making it impossible to calculate an effect size for the coaching intervention.

For those studies that were included in the meta-analysis, the effect sizes were split into two categories dependent on the source of the rating of performance: self versus others, and separate meta-analyses were conducted for each category. The results of these meta-analyses showed the improvement in skill or job performance after a coaching intervention was a moderate-to-large effect, although it was perceived as a substantially larger effect of 1.27 when rated by self, compared to a moderate-to-large effect of .50 as rated by others. However, in addition to the small number of studies included in this meta-analysis the variability between the effect sizes found in these studies was rather extreme, ranging from .02 to 1.98 from self-ratings and .06 and 1.83 for other-ratings. These results would suggest that coaching, as an intervention, ranges from completely ineffective to a very large and impressive effect size indicating large differences. These results highlighted that there was a great need for more methodologically rigorous studies in the coaching space and that it would a be a number of years before an adequate reserve of studies would be available for a conclusive meta-analysis of the effectiveness of coaching [10].

### Coaching: Current Perspectives

Although some early work in the coaching space did consider outcomes, evaluation and the effectiveness of the intervention the number of studies were limited. However, since then more coaching studies investigating its efficacy have been published. A more recent meta-analysis [18] faired much better in terms of quantitative studies that meet the inclusion criteria: 18 studies, however a considerably larger number of studies were initially identified: 107 studies. Their inclusion criteria were centred on the Grant [19] definition of life coaching: ‘a collaborative solution-focused, result-oriented, systematic process in which the coach facilitates the enhancement of life experience and goal attainment in the personal and/or professional life of normal, non-clinical clients’ (p. 254). Although, the final number of studies included in this review was three times the number included by De Meuse et al. [17] it is still rather a low number of studies for a conclusive meta-analytic procedure [20]. Theeboom et al. [18] focused their meta-analysis on answering the question ‘Does coaching work?’ They included studies that investigated personal and business coaching and examined the impact of these interventions on individual-level outcomes: performance/skills, well-being, coping, attitudes and self-regulation, in an organisational context.

Their results showed that coaching had a positive and significant impact on all of these individual-level outcomes with effect sizes ranging from .43 for coping and .74 for self-regulation. The effect of coaching on performance/skills was found to be .60, however, removal of single study that was identified through sensitivity analysis saw this effect size drop considerably to .19, which is considered a small effect. Further investigation of the heterogeneity of effect sizes between studies found that study design had a substantial impact on the magnitude of the effect size for some of the outcome categories. In addition, the authors examined the impact of number of coaching sessions on the outcomes and found that greater sessions did not necessarily result in greater positive effects and in some outcomes more sessions was found to potentially be detrimental. For example, higher effects were found for fewer sessions for performance/skills and work attitudes. However, it is unclear whether this is a result of the type of coaching intervention or a consequence of the magnitude of the problem addressed by the coaching intervention.

Although the authors focused their investigation of coaching effectiveness in organisational contexts they did include personal/life coaching interventions as well as coaching studies conducted in a university setting with undergraduate students. Furthermore, they included studies that utilised alternative mechanisms for coaching such as peer and online coaching. Additionally, they excluded those studies that used coaching in tandem with other organisational development programs, such as leadership development. Although, this exclusion is important when isolating the specific effects of coaching it does limit how applicable this research is to organisations in which leadership development may occur in tandem with coaching. Finally, this meta-analysis focused its investigation on outcomes at the individual-level, and although these are relevant for organisations and organisational performance, there was no specific investigation of organisational-level outcomes.

A further meta-analysis [21] has been conducted to examine the impact of coaching in an organisational setting. This meta-analysis consisted of 17 studies and investigated the effectiveness of coaching on outcomes that were split into affective, skill-based and individual-level outcomes. The results showed positive effect sizes, which ranged from small to large: .26 for skill-based outcomes, .46 for affective outcomes and 1.15 for individual-level outcomes. Although the researchers had originally set out to examine the impact of coaching on team and organisational outcomes as well as the individual-level none of the studies that matched criteria for inclusion in the meta-analysis covered variables outside of the individual. This is an area within coaching effectiveness research that is severely overlooked. There is little insight into how coaching might potentially impact the individuals who work closely with the coachee.

### Measuring Effectiveness: Key Debates

#### Coaching outcome measures

The diversified outcomes addressed in coaching make coaching objectives inherently incomparable as an outcome measure. Following this, a number of different outcome measures have been investigated in coaching effectiveness studies, including but not limited to: job satisfaction, job performance, self-awareness, self-efficacy, positive affect, depression, anxiety, resilience, hope, autonomy and goal attainment (see Theeboom et al., [18] for a list of all the outcome measures investigated in their meta-analysis). Disparate outcome measures aside there is also a great concern around research design and methodology of coaching studies. Of the 107 studies initially identified by Theeboom and colleagues [18], 69 of them were not included in the analysis because of the poor study design, such as cross-sectional design, and lack of quantitative analysis.

This concern around research methodology has been an on-going issue with the coaching literature having also been mentioned in the review by Kampa-Kokesch and Anderson [12]. However this issue is not isolated to the coaching research but has been faced by other domains within organisational, management and psychological research. The use of inferior methodological design in these domains is a result of the restraints associated with researching organisations. It is inherently difficult to get access organisation and their people. These are known limitations of quantitative organisational research and there are several known methodological shortcomings within the organisational/management research field that have been highlighted by various authors (e.g. Antonakis, Bendahan, Jacquart, and Lalive [22] and Bono and McNamara [23]).

Passmore and Fillery-Travis [10] discussed in their review, there is an evolution associated with the development of knowledge in a new field and different phases of this evolution have different characteristics that influence the type of research that is conducted. For example, this evolution often starts with an exploration phase, which is focused on defining the area or boundary of study, exploration and sharing of processes between practitioners and consists of more theoretical papers. The next phase consists of case studies and small empirical studies that test the theories, measures and models underlying the area. Following this, larger quantitative studies with randomised control groups are conducted with large sample sizes and then meta-analyses are conducted across these studies. The final stage then examines what factors impact the effectiveness of the phenomena, such as mediators and moderators. In their review, Passmore and Fillery-Travis [10] found coaching research to be in the second phase but towards the end of this phase as the number of randomised control group studies was increasing and a meta-analysis had been attempted. Given that there are over 20 years of published papers in the area it is now clearly time for a careful systematic review of the literature.

#### Return on investment (ROI)

One possible organisational-level outcome, ROI specifically financial ROI, is an area within coaching evaluation that has caused quite divergent opinion amongst all the stakeholders of coaching. ROI is seen by numerous organisations as a comparison measure that can be used with disparate interventions or processes to create a comparable tangible value for them [24]. Furthermore, coaching can be a rather expensive intervention: implementation of a six-month coaching intervention within an organisation setting can cost anywhere between $15,000 to $75,000 and does not include the opportunity cost of the executive’s time spent with the coach during working hours [17]. With such a costly intervention, it is understandable that organisational coordinates of coaching, such as human resources directors, need evidence of its effectiveness and the impact of coaching on distal organisational outcomes.

De Meuse and colleagues [17] conducted a content analysis on the 13 studies that were not included in the meta-analysis and found there were a number of authors that had investigated ROI and had found values that ranged from 600% to 700%. However, the technique for calculation of these figures was found to be rather subjective and tenuous. These results, along with numerous other papers (e.g., Grant [5] and Lawrence and Whyte [24]), highlight that ROI may not be the most accurate or useful measure for evaluating coaching in an organisational setting. The difficulty is rooted in the coaching intervention itself, coaching is usually a customised, one-on-one intervention [14].

Coaching outcomes and goals differ from session to session and from individual to individual as well as during the engagement. Kauffman and Coutu [25] found from 140 coaches surveyed that the overwhelming majority of them, 132, said that the focus of their coaching session shifted during the engagement and cited a number of reasons why: deeper goals, natural evolution, self-awareness, coaching relationship and circumstances. In addition, context and environmental factors differ from one intervention to the next, even within the same organisation, one individuals supervisor might be supportive of coaching while another is not. Outside of the client and the context, the coaches also vary significantly, for example in terms of their training, their background and the techniques and models that they use.

Additionally, it is incredibly difficult to isolate the specific impact of a coaching intervention on an individual or group of individual’s performance let alone any distal impacts within the organisation. The links between coaching and monetary changes within an organisation are likely to be complex and as yet there is no reliable way to measure or calculate the benefits of coaching in terms of a financial ROI estimation.

Furthermore, Lawrence and Whyte [24], who interviewed purchasers of executive coaching, found that only a small number (14%) used ROI when evaluating the effectiveness of their coaching intervention. However, the purchasers discussed ROI as being a tool that accompanied other evidence of the outcomes of coaching. Additionally, these purchasers of coaching described other metrics such as staff engagement and retention as being more important metrics to evaluate the effectiveness of coaching. On the other hand, Grant [5](2012) suggests a more extreme move away from traditional organisational metrics to a more holistic, person-centred measure of effectiveness of coaching focused on well-being and engagement. There is still continued debate around ROI and the appropriate tools and outcomes that should be used to measure the effectiveness of coaching. However, it is clear that more distal outcomes of coaching related to organisations is needed such as the impact on subordinates’ satisfaction, retention and performance, the impact on relationships or communication between different teams and the impact on the overall organisational or team culture.

#### Moderators and mediators of effectiveness

The final stage of knowledge evolution as described by Passmore and Fillery-Travis [10] relates to the moderators and mediators that impact the effectiveness of coaching. Baron and Kenny [26] define a moderator as a variable that influences the direction and/or magnitude of the relationship between a predictor and a dependent variable. On the other hand, they define a mediator as a variable, which accounts for the relationship between a predictor and a dependent variable. It can be described as a variable that sits in between the predictor and dependent variable and acts as a conduit of the relationship. Leveraging the psychotherapy space, the working alliance between a coachee and coach could form a potential moderator or mediator between client attributes and coaching outcome [27]. Furthermore, individual differences, such as personality, emotional intelligence or IQ, could play moderating roles between the type of coaching intervention and its effectiveness.

Although coaching research remains nascent in this arena a number of contributing factors have been recommended for further exploration. These factors have been identified by looking at other disciplines that are believed to align with coaching, such as counselling and therapy. One seminal paper [28] in this area compared the active ingredients from psychotherapy, which are based on decades and decades of research, to the authors’ own encounters in executive coaching. The first factor described is the client themselves, in terms of readiness and motivation, and the unique circumstances related to the client, such as client experience and environment. This is believed to be the largest contributor of the outcome. The next one is the relationship between the client and coach and the final two are the client’s expectations and the theories and techniques utilised. Following this paper, a number of researchers have begun to explore these “active ingredients” in experimental conditions to find evidence to support this theory (e.g. De Haan, Duckworth, Birch and Jones [29]).

In addition to examining the effectiveness of coaching, Jones et al. [21] explored some of the potential moderators that may influence the effectiveness of coaching. They examined research design, multisource feedback, the format of coaching, the schedule of coaching and the type of coach. They found no effect for research design, longevity of coaching, number of coaching sessions and format of coaching. The results related to number of coaching sessions and coaching effectiveness mirrors that found by Theeboom et al. [18], however the results for research design moderating the effect of coaching was opposing. This may be because Jones et al. examined the effects of research design across all of the outcome categories whereas Theeboom et al. [18] examined the effects on each outcome category separately. This could allude to the fact that the effects of certain outcomes could be more influenced by study design than others. However, this is yet to be explored.

Additionally, Jones et al.’s [21] results did show more positive effects of coaching by an internal coach as apposed to an external coach and for coaching without multisource feedback. However, with only a small number of studies in this analysis it is difficult to make conclusive judgments about these moderators. Examining moderators with such a limited number of studies can produce potentially erroneous results. For example, in the Jones et al. [21] meta-analysis all of the studies that utilised multisource feedback also used an external coach. It is therefore unclear whether the lower effect size for studies using multisource feedback is attributable to the use of multisource feedback or the use of an external coach. This could potentially have confounded the effects of coaching on the outcomes assessed. The subgrouping of studies to investigate moderators can lead to confounding of variables if there are not enough viable studies to be included in the analysis [30].

Coaching is such a complex process it is likely that a number of factors contribute to its success but it is also like these factors may interact with one another as well. However, much of this remains unexplored. One thing that is clear is that there are not enough experimentally rigours studies in the coaching literature to warrant definitive claims about moderating effects as yet. Furthermore, the coaching efficacy literature encompasses a number of different outcome measures. The use of these different outcome measures, such as self-awareness, goal attainment or other individual-level outcomes, result in huge variability of effect sizes as they are completely different constructs. Therefore, it is utterly meaningless to compare the effect sizes of different constructs across studies. One study [31] has explored multiple individual-level outcome measures and their results show the magnitude of the variability that is seen across coaching outcome measures.

### Present Study

#### Rationale

**Fig 1:**
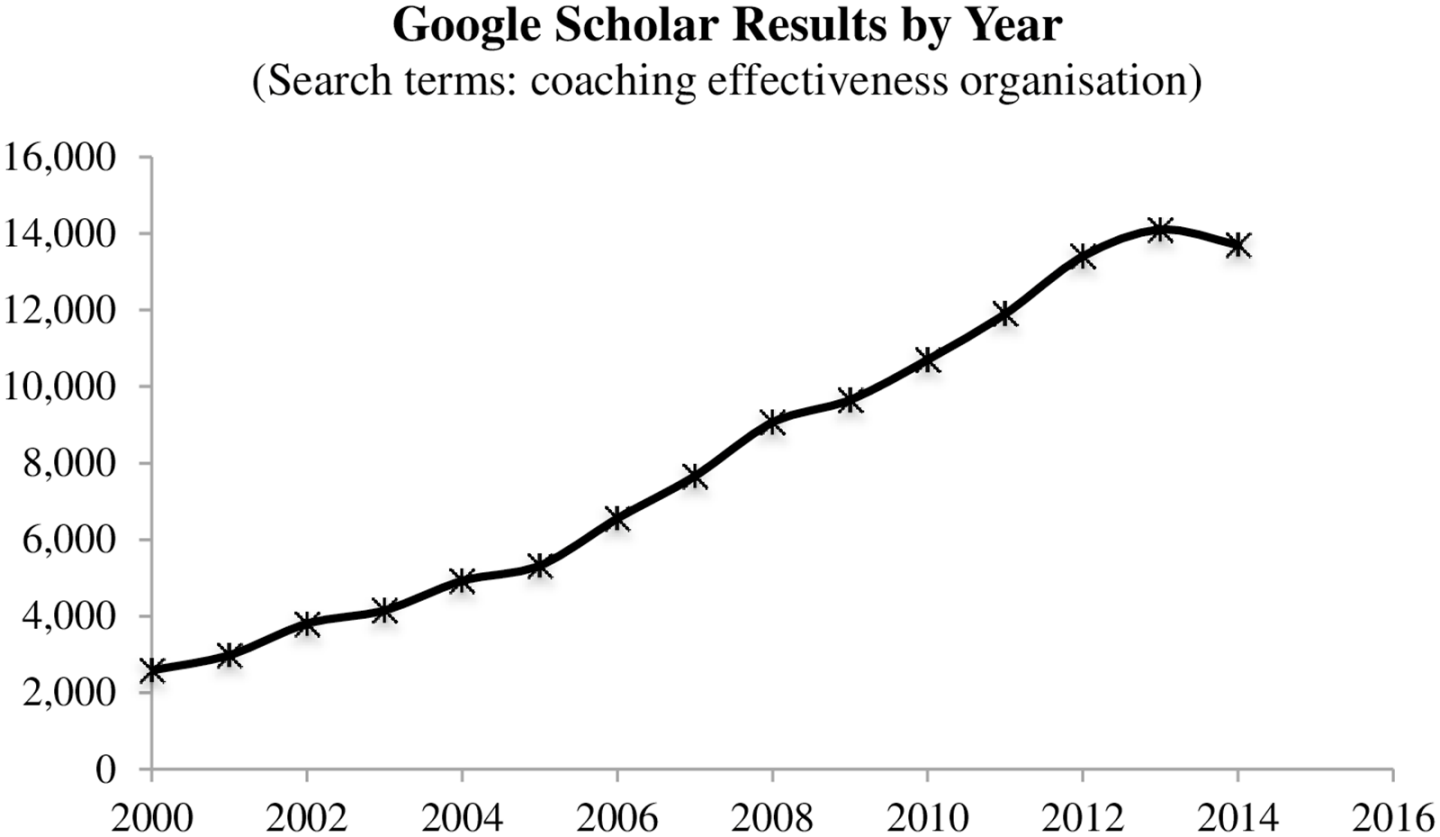
Graph Showing Google Scholar Results by Year for the Following Search Terms: Coaching, Effectiveness and Organisation.

The number of studies and papers discussing and investigating coaching has increased steadily over the last decade. Fig 1, shows that a simple examination of Google Scholar results highlights this increase. Although a number of reviews into the effectiveness of coaching have been conducted over the last decade (e.g., Ely et al. [14], Grant, Cavanagh, and Parker [32], Joo [2] and Passmore and Fillery-Travis [10]). This review specifically examines individual and distal outcomes that are relevant for the organisation. It also looks to review the evidence related to the mechanisms that potentially underlie the effectiveness of coaching, by examining the literature investigating moderators and mediators of coaching effectiveness.

Understanding the moderators and mediators of the effectiveness of coaching can help answer a number of questions that organisations have about coaching, such as: are particular types of people more suited to being coached, at what level of executive is coaching most effective at improving organisational performance, are there particular types of problems that are more suited to coaching, can coaching have negative impact on organisational performance, what type of coach would suit your employees and does the relationship between the coachee and the coach impact the effectiveness of the intervention? The exploration of underlying mechanisms is important for organisations because the results can enable organisations to be selective in their implementation of coaching and ensure that the coaching intervention results in maximum effectiveness.

As discussed above, a number of issues related to the coaching literature have been highlighted in previous reviews, such as the suitability of variables as coaching outcomes and research methodology, an additional goal of this review is to track the progress of coaching literature related to these issues. Consequently, those issues that are still unaddressed by the coaching literature will be exposed. Additionally, research areas and focus for the next decade of coaching literature will be considered.

This review differs from recent meta-analyses such as Jones et al. [21] and Theeboom et al. [18] by examining a wider array of coaching effectiveness studies with the aim the shed light on the distal and organisational impacts of coaching alongside the impact on coachees. Meta-analyses are far more stringent in the requirements of underlying studies than a review, which is why these two previous meta-analyses have only included 17 and 18 studies respectively.

#### Objectives

To examine whether coaching interventions are effective, we reviewed quantitative studies, encompassing both control group and non-control studies that assessed the efficacy of business, executive and leadership coaching interventions in employed adults in organisational settings. In addition, we evaluated the evidence pertaining to variables that potentially moderate or mediate the effectiveness of coaching interventions within this population.

## Method

**Fig 2:**
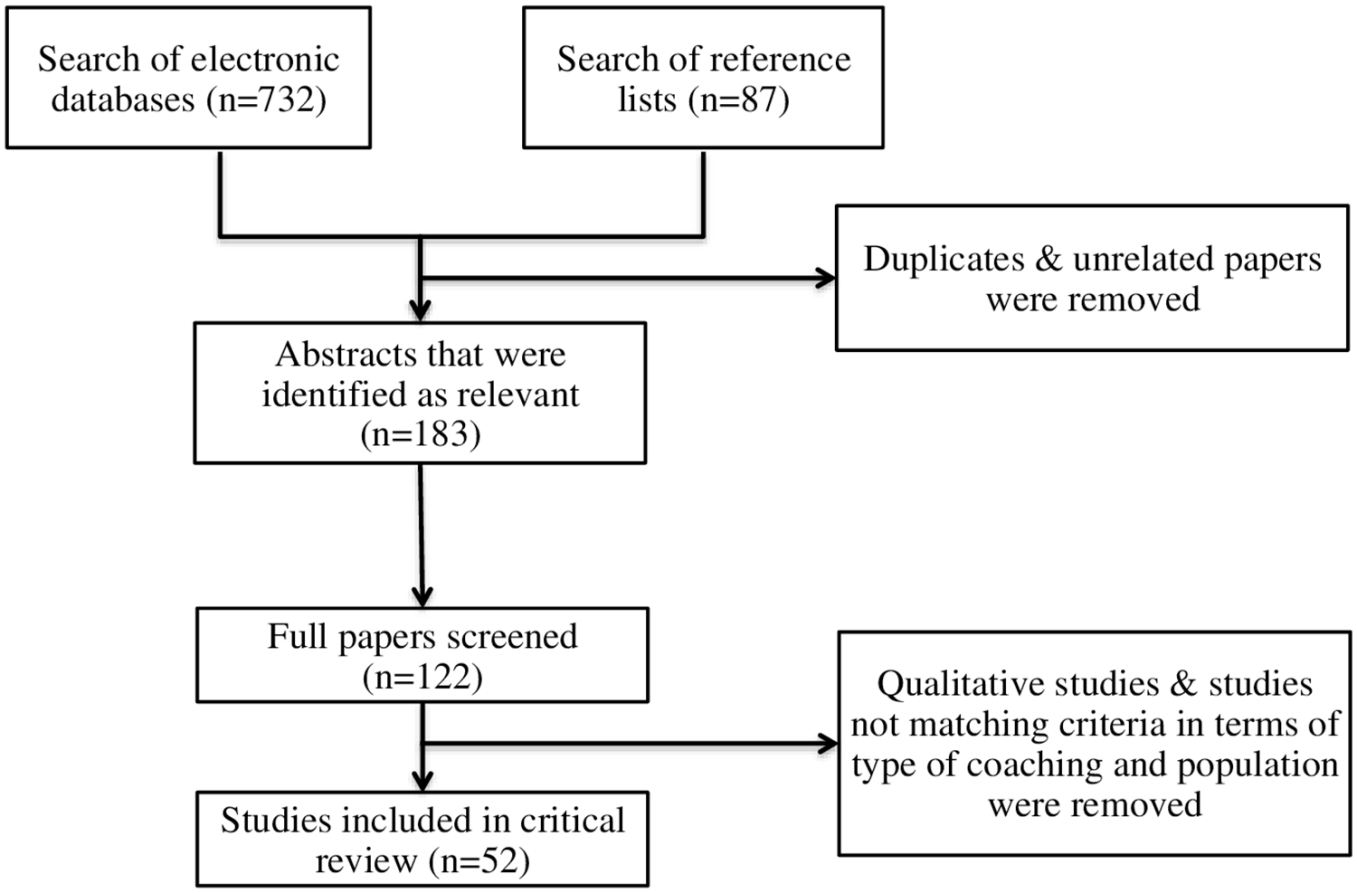
Diagram Depicting Systematic Search Style Implemented in This Review.

Electronic databases (e.g., PsycINFO, Scopus and Google Scholar) and reference lists were searched for relevant papers published since 2003, using the following key words: leadership coaching, business coaching, executive coaching, work place coaching, developmental coaching, coaching effectiveness, coaching efficacy and coaching evaluation, coaching outcomes, and effects of coaching. A systematic search strategy was used to assess inclusion of studies in this review (see Fig 2). Firstly, we searched for articles that included quantitative data on the effectiveness of coaching or the mechanisms that contribute to the effectiveness of coaching. Furthermore, only those studies that were conducted in an organisational setting or utilised coachees in full-time or part-time employment were used. Coaching studies utilising students or university settings were excluded. Although, university demands can be incredibly high and academic performance can be a valuable outcome. Student environments do not effectively mimic the requirements of an organisation. Additionally, coachees in an organisational setting often have many years of work experience that contribute to their unique circumstances and, as discussed above, this forms one of the active ingredients in the effectiveness of coaching. Furthermore, the majority of students, especially undergraduate students, have limited or no experience of working in an organisational environment.

Also, we only included studies that utilised a professional coach, both external and internal, but who did not have any managerial supervision over the coachee. Coaching from a position of authority, often referred to as manager-as-coach in the literature, is a prevalent phenomenon and its exclusion is no reflection on its effectiveness in an organisational setting. However, having supervisory power or influence over a coachee may impact the focus of the coaching sessions. Research in the manager as a coach space has found it to be a useful tool for improving the performance of employees, influencing employee behaviour and working towards mutually acceptable goals [33]. On the other hand, coaching from a non-supervisory individual is completely focused on the coachee and their needs in terms of development areas, which may ultimately align with the organisation but it is the coachees’ decision to make. Further, the relationship between coachee and manager as a coach is likely to be different to that of an impartial coach especially related to closeness and openness and as other talking therapies have demonstrated this relationship can have considerable impact on the effectiveness of coaching. This distinction, in terms of focus and also the alternative dynamic that is likely to exist between manager and employee versus coach and employee, is why any studies pertaining to manager as a coach was excluded.

Furthermore, this review focuses on coaching explicit to an organisational environment; therefore, studies pertaining to health, life or sports coaching were excluded. Although, there is considerable commonality between business coaching and life coaching in terms of the facilitative nature, non-clinical populations and working towards a client specified goal [34], the overall focus of the sessions in business coaching is often, though not always, centred around performance or problems in the workplace, however, personal issues may be addressed if they are impacting the coachee’s performance. Whereas life coaching is often centred on more personal aspects that may occasionally be the same as those addressed in business coaching but they may also be completely separated from the workplace.

Further, alternative coaching mediums, such as online coaching or team or peer coaching, were excluded. Although, online coaching is currently a growing trend in the coaching sphere driven by technology advancements and research investigating the effectiveness of online coaching is emerging (e.g. Poepsel [35]). It is still a nascent and evolving intervention and it is unclear how an online relationship, as opposed to a face-to-face relationship, may impact the formation and depth of relationship between coachee and coach, which is believed to be a key active ingredient in the effectiveness of coaching. Research in other domains has found that online relationships, such as those on social media sites, can have the same depth and connectedness as in-person interactions [36]. Furthermore, an examination of the research pertaining to the effectiveness of online psychotherapeutic interventions [37] found a medium positive effect however the results also showed that the effectiveness of online interventions differed by therapeutic approach so it is not clear whether this evidence naturally translates to coaching as there are a number of different techniques utilised in coaching interventions.

In terms of peer coaching, one study [38] that examined the relative effectiveness of peer coaching and external coaching in a student sample found that external coaching proved to be more effective, in terms of performance and satisfaction, over peer coaching as the coach was perceived to be more credible in external coaching. Although these alternative methods of coaching are not included in this review, online, peer and team coaching will require their own separate reviews in due course. Following this, a comparison of effectiveness across coaching mediums will prove insightful. Studies that used individual, face-to-face coaching in combination with group or telephone coaching were, however, included in this review.

Finally, studies that included a coaching intervention in tandem with other developmental interventions, such as classroom-training, leadership or management development or multisource feedback, were included in this review because very often these types of developmental interventions and training are part in parcel in the majority of organisations that have any sort of employee development or performance management program. From an academic standpoint, it would be ideal to have a control group to isolate the specific effects of coaching above and beyond another intervention; however, from an organisational perspective demonstrating the value of coaching in combination with existing prevalent interventions is value in itself [17].

## Results

As Fig 2 shows, 52 studies met the inclusion criteria and were examined in this review. The majority of studies involved in this review were from peer-reviewed journals, although four dissertation studies [39–41] and two conference papers [42, 43] were also included. Table 1 provides an overview of the experimental attributes of the studies, including the type of analysis utilised, and the categories of outcomes examined and gives an overview of the range of participants and coaching sessions investigated. It is clear that the number of participants, in terms of coachees, coaches, control groups and other-raters, such as supervisors, subordinates and peers, varied considerably as well as the number of coaching sessions utilised, ranging from one to twelve.

**Table 1:** Overview of Experimental Attributes Across Studies.

**Experimental design**	**No. of studies**
Pre- & post-design	30
Retrospective	16
Control group	24
Longitudinal	5
**Rating sources**	**No. of studies**
Self-report	40
Other-report	24
Multisource	12
Objective	3
**Outcome examined**	**No. of studies**
Individual	32
Organisation	18
**Mechanism**	**No. of studies**
Coachee	12
Coach	12
Coachee-Coach relationship	9
**Participants across studies**	**Range (min—max)**
Coachees	1–638
Coaches	1–96
Control group	6–1842
Other-raters	28–242
Coaching sessions	1–12

### Research Design

In terms of experimental attributes, even though the bulk of the studies came from peer-reviewed journals the experimental procedures varied considerably. Over half of the studies involved pre and post intervention assessment (*n* = 30) and just under half had a control group (*n* = 24). However, a significant number, nearly one third of all the studies examined, used only a retrospective design (*n* = 16). Additionally, just a handful of studies, five of them [40, 44–46] had some sort of additional follow up assessment a number of months after the post assessment to examine the longitudinal effects of coaching.

Although nearly all of the studies used questionnaire or interview based assessment a couple of studies used more novel approaches that could aid future research design. For example, Ianiro, Schermuly and Kauffeld [47] used video recordings of the first coaching sessions to measure the affiliation and dominance behaviour of the coach and client. In another study, Perkins [48] observed the meetings of coaching clients in order to assess changes in meeting behaviours after implementation of a coaching intervention.

The majority of studies had some aspect of self-report to assess the outcomes of the coaching intervention, although 20 of those studies only used self-report measures to assess the effectiveness of coaching. Most of the studies in this review focused on the effectiveness of coaching on the individual (i.e., the coachee or client) (*n* = 32) but 18 of the total studies did investigate some sort of organisational impact. This includes those studies that used changes in multisource ratings and also measured the effect of coaching on others outside of the coachee within the organisation, such as subordinates. Those studies that explored mechanisms of effectiveness can be grouped into three areas: those characteristics related to the coachee, the coach and the relationship between the coach and coachee. Before exploring these mechanisms in more detail we look at the overall effectiveness of coaching on the individual and those who work around them. Table 2 gives an overview of the experimental attributes of each study included in this review and Table 3 gives a detailed summary of the outcomes and variables investigated by each study.

**Table 2:** Overview of experimental and analytical procedure across studies.

Author	Year	Participants				Outcome		Mechanism			Data Collection				Analysis
		Coaches	Coachees	Control Group	Other-raters	Ind	Org	Coachee	Coach	C-C Relationship	Pre	Post	During	Longitudinal	
Gan & Chong [79]	2015		172							Y		Y			multiple regression
Sonesh, Coultas, Marlow, Lacerenza, Reyes & Salas [55]	2015	17	90		Y	Y		Y	Y	Y		Y			correlation & multiple regression
Biggs, Brough & Barbour [68]	2014			Y	Y*		Y				Y	Y			multiple regression
Hoven, Ford, Willmot, Hagan & Siegrist [44]	2014		638	Y		Y						Y		Y	multivariate regression
Ladegard & Gjerde [49]	2014	7	24	Y	Y	Y	Y		Y		Y	Y			t-test, multiple regression
Jones, Woods & Hutchinson [70]	2014	-	30					Y				Y			correlation
Gatling & Harrah [77]	2014	96	-						Y			Y			CFA, ANOVA, multiple regression
Grant [57]	2014	49	49			Y				Y	Y	Y			correlation, t-test
Mackie [46]	2014	11	31	Y	Y	Y	Y		Y		Y	Y	Y	Y	ANCOVA, mixed ANOVA
O’Connor & Cavanagh [54]	2013	8	20	Y	Y	Y	Y				Y	Y			ANOVA, t-test, SNA
Ianiro, Schermuly & Kauffeld [47]	2013	32	32		Y	Y			Y	Y	Y	Y			simple & multiple regression
Smith & Brummel [63]	2013	-	30		Y	Y		Y				Y			correlation, ANOVA
De Haan, Duckworth, Birch & Jones [29]	2013	34	156		Y			Y	Y	Y		Y			correlation, t-test, multiple regression
Grant [31]	2013	-	31			Y					Y	Y			t-test
Nieminen, Smerek, Kotrba & Denison [66]	2013	-	227	Y	Y	Y	Y				Y	Y			repeated measures ANOVA
Bozer & Sarros [60]	2012	68	72	Y	Y	Y	Y				Y	Y			two-way repeated measures ANOVA
Bozer, Sarros & Santora [75]	2013	68	72	Y	Y	Y	Y	Y			Y	Y			hierarchical regression
Bozer, Sarros & Santora [74]	2014	68	72	Y	Y	Y	Y		Y		Y	Y			two-way repeated measures ANOVA
Bozer, Joo & Santora [80]	2015	68	72	Y	Y	Y	Y			Y	Y	Y			multiple regression
Vidal-Salazar, Ferrón-Vilchez and Cordón-Pozo [69]	2012	-	20	Y	Y		Y					Y			Wilcoxon-Mann-Whitney test
Bright & Crockett [62]	2012	1	73	Y		Y					Y	Y			t-test
Blackman & Moscardo [42]	2012	-	114					Y	Y			Y			t-test, ANOVA, multiple regression
Crompton [39]	2012	-	100	Y		Y	Y		Y			Y			structural equation modelling
Ladegård [45]	2011		56			Y					Y	Y		Y	t-test, multiple regression
De Haan, Culpin & Curd [73]	2011	-	30			Y		Y	Y		Y	Y			t-test, multiple regression
Scriffignano [76]	2011	-	110					Y				Y			correlation
Richardson [41]	2010	5	6	Y	Y	Y					Y	Y			t-test
Kochanowski, Seifert & Yukl [67]	2010	1	12	Y	Y		Y				Y	Y			two-way repeated measures ANOVA
Leonard-Cross [4]	2010	-	61	Y		Y						Y			t-test
Cerni, Curtis & Colmar [65]	2010	1	8	Y	Y		Y				Y	Y			t-test
Grant, Green & Rynsaardt [53]	2010	-	23	Y	Y	Y	Y				Y	Y			two-way repeated measures ANOVA
Grant, Curtayne & Burton [52]	2009	-	41	Y		Y					Y	Y			two-way repeated measures ANOVA
Baron & Morin [27]	2009	24	73		Y	Y		Y	Y	Y	Y	Y			t-test, hierarchical regression
Baron, Morin & Morin [78]	2011	24	73		Y	Y			Y		Y	Y			ANCOVA
Perkins [48]	2009	1	21		Y		Y	Y			Y	Y			correlation, multiple Wilcoxon sign rank test
Moen & Skaalvik [97]; Moen & Federici [98]; Moen & Federici [50]	2009;2012;2012	3	11	Y		Y					Y	Y			t-test, ANCOVA
Stewart, Palmer, Wilkin & Kerrin [72]	2008	-	110					Y				Y			correlation, multiple regression
Yu, Collins, Cavanagh, White & Fairbrother [56]	2008	1	10			Y					Y	Y			Wilcoxon sign rank test
Finn (Study 1) [40]	2007	5	7	Y		Y					Y	Y	Y	Y	two-way mixed factorial ANOVA
Finn (Study 2) [40]	2007	9	11	Y	Y	Y	Y				Y	Y	Y	Y	two-way mixed factorial ANOVA
Bowles, Cunningham, De La Rosa & Picano [61]	2007	2	30; 29		Y	Y	Y	Y			Y	Y			t-tests, chi-square non-parametric analyses, & multiple regression
Orenstein [99]	2006	1	1		Y		Y					Y			t-test
Evers, Brouwers & Tomic [51]	2006	-	30	Y		Y					Y	Y			ANOVA
Scoular & Linely [71]	2006	14	120		Y					Y		Y			n/a
Jones, Rafferty & Griffin [64]	2006	5	11	Y		Y					Y	Y	Y		Repeated measures ANOVA
Toegel & Nicholson [43]	2005	-	89		Y	Y	Y			Y	Y	Y			t-test, hierarchical regression
Gyllensten & Palmer [58]	2005	-	16	Y		Y					Y	Y			two-way mixed factorial ANOVA
Gyllensten & Palmer [59]	2005	-	62	Y		Y						Y			multiple regression
Smither, London, Flautt, Vargas & Kucine [16]	2003	-	400	Y	Y	Y	Y				Y	Y			MANCOVA, hierarchical regression
Luthans & Peterson [15]	2003	1	20		Y	Y	Y				Y	Y			t-test, paired t-test

*Note*. C-C = Coachee-coach.

*This study examined the effects of coaching on the subordinates of coachees (n = 146).

**Table 3:** Overview of variables investigated and indication of results found across studies.

Author	Year	Individual	Organisation	Mechanism measured
Gan & Chong [79]	2015			**Rapport,** Trust, **Commitment**, Coach-Coachee Match
Sonesh, Coultas, Marlow, Lacerenza, Reyes & Salas [55]	2015	**Insight, Goal attainment**		Coachee Motivation, Goal difficulty (MOD), *Working alliance* (MED), *Information sharing* (MED), Coaching behaviour, Coach’s psychological mindedness
Biggs, Brough & Barbour [68]	2014		**Subordinate work engagement, Subordinate job satisfaction**, Subordinate turnover intentions, Subordinate psychological strain	Subordinate perception of job demands (MED), **Subordinate perception of strategic alignment (MED), Subordinate perception of work-culture support (MED),** Subordinate perception of supportive leadership (MED)
Hoven, Ford, Willmot, Hagan & Siegrist [44]	2014	**Successful employment, Sustained employment**		**Age (MOD), Ethnicity (MOD), Educational level (MOD)**
Ladegard & Gjerde [49]	2014	**Leader efficacy, Leader's trust in subordinates**	Psychological empowerment (subordinates)**, Turnover intention (subordinates)**	**Coach facilitative behaviour**
Jones, Woods & Hutchinson [70]	2014			*Coachee personality*
Gatling & Harrah [77]	2014			**Coach authentic leadership**
Grant [57]	2014	**Goal attainment, Psychological well-being,** Depression, **Anxiety, Stress, Self-insight**		**Autonomy Support,** Satisfaction of the C-C relationship, **Similarity to ideal relationship, Goal-focused relationship** (However, once controlling for goal-focused relationship the other components became non-significant)
Mackie [46]	2014	**Transformational leadership (multirater)**	**Transformational leadership (multirater)**	**Strength-based methodology**
O’Connor & Cavanagh [54]	2013	**Well-being, Goal attainment, Transformational leadership (multirater), Quality of communication (self)**	**Quality of communication (others) [NEGATIVE],** *Well-being of those close to coachee*, Quality of communication across organisation	
Ianiro, Schermuly & Kauffeld [47]	2013	**Goal attainment**		Affiliation of coach, **Dominance of coach, Interpersonal similarity (this also predicted quality of relationship)**
Smith & Brummel [63]	2013	**Competency change**		**Coachee involvement, Developability of competency**, *Individual development plan*
De Haan, Duckworth, Birch & Jones [29]	2013			Coachee personality, **Coachee self-efficacy,** Mismatch of coachee/coach personality, **Range of coaching techniques, Quality of relationship, Working alliance (client) (MED) (fully mediates between self-efficacy and coaching outcome and partially mediates between techniques and coaching outcome),** Working alliance (coach)
Grant [31]	2013	**Goal attainment, Solution-focused thinking, Change readiness, Leadership self-efficacy, Depression,** Anxiety, Stress, **Resilience,** Workplace satisfaction		
Nieminen, Smerek, Kotrba & Denison [66]	2013	*Leadership behaviours (multisource)*	*Leadership behaviours (self)*, Leadership behaviours (direct reports, peers & supervisors)	
Bozer & Sarros [60]; Bozer, Sarros & Santora [75]; Bozer, Sarros & Santora [74]; Bozer, Joo & Santora [80]	2012; 2013; 2014; 2015	Self-awareness, **Career satisfaction**, Job affective commitment, Job performance	Job performance (self), Job performance (supervisor), Task performance (supervisor)	*Coachee pre-training motivation (MOD)*, *Coachee feedback receptivity (MOD)*, *Coachee learning goal orientation Coachee developmental self-efficacy*, *Coach’s academic background in psychology*, *Coach’s credibility*, *Gender similarity*, *Perceived similarity*
Vidal-Salazar, Ferrón-Vilchez and Cordón-Pozo [69]	2012		*Effectiveness of business improvement plan implementation*	
Bright & Crockett [62]	2012	*Performance*		
Blackman & Moscardo [42]	2012			*Coachee demographics*, Coach features, Coaching process, **C-C similarity as rated by coachee**, *completion of coaching*, organisational support, **coachee goals and effort**
Crompton [39]	2012	**Locus-of-control internal, Locus-of-control external, Self-efficacy**	**Firm growth (objective)**	*Coaches' role*, *Session focus*, *Coaching results*, Coaching satisfaction
Ladegård [45]	2011	**Stress**		Insight (MED), **Planning skills (MED), Job demand (MED), Job control** (MED), **Social support** (MED)
De Haan, Culpin & Curd [73]	2011	**Helpfulness**		**Coachee learning styles,** *Coach behaviours*
Scriffignano [76]	2011			*Coachee goal orientation*
Richardson [41]	2010	**Working alliance,** Goal attainment, Life satisfaction		
Kochanowski, Seifert & Yukl [67]	2010		*Influence of behaviour (subordinates)*	
Leonard-Cross [4]	2010	**Self-efficacy,** Anxiety, **Optimism, Work Satisfaction, Self-awareness**		
Cerni, Curtis & Colmar [65]	2010		**Multifactor leadership questionnaire (subordinates)**	
Grant, Green & Rynsaardt [53]	2010	**Goal attainment,** Depression, Anxiety, **Stress, Workplace well-being, Resilience, Leadership styles (multisource)**	**Leadership styles (self)**, Leadership styles (others)	
Grant, Curtayne & Burton [52]	2009	**Goal attainment,** *Depression*, Anxiety, *Stress*, **Resilience, Workplace well-being**		
Baron & Morin [27]; Baron, Morin &Morin [78]	2009; 2011	**Self-efficacy**		*Coachee motivation to transfer, *Coachee perception of supervisor support, *Coach relational skills, *Coach communication skills, *Coach's self-efficacy with regard to facilitating learning & results, *No of coaching sessions, Working alliance (MED between no. of coaching sessions and outcome, *correlates of working alliance), Coach’s under-/over-/accurate-estimation of working alliance
Perkins [48]	2009		**Meeting behaviours**	Coachee age, Coachee verbal IQ score
Moen & Skaalvik [97], Moen & Federici [98], Moen & Federici [50]	2009; 2012; 2012	*Goal setting*, **Self-efficacy**, *Attribution*, *Self-determination*		
Stewart, Palmer, Wilkin & Kerrin [72]	2008			*Coachee personality*, *Coachee self-efficacy*
Yu, Collins, Cavanagh, White & Fairbrother [56]	2008	**Goal attainment, Proactivity, Motivation, Core performance behaviour,** Well-being, Meta-cognition		Goal type
Finn (Study 1) [40]	2007	*Self-efficacy*, *Developmental support*, *Positive affect*, *Openness to new behaviours*, *Developmental planning*		
Finn (Study 2) [40]	2007	*Transformation Leadership (multisource)*, *Self-efficacy*, *Developmental support*, *Positive affect*, *Openness to new behaviours*, *Developmental planning*	*Transformational Leadership* (Self rating, Supervisor rating, **Peer rating)**	
Bowles, Cunningham, De La Rosa & Picano [61]	2007	*Leader competency ratings***, Coachee performance (objective data)**		**Coachee buy-in, Coachee level (executive vs middle manager)**
Orenstein [99]	2006		**Other-ratings of behaviours related to coaching objectives, Other-ratings of behaviours indirectly related to coaching objectives**, Other-ratings of control behaviours	
Evers, Brouwers & Tomic [51]	2006	*Outcome expectations*, *Self-efficacy beliefs*		
Scoular & Linely [71]	2006			*Coachee and coach match/mismatch on personality*, Goal versus non-goal setting
Jones, Rafferty & Griffin [64]	2006	**Managerial flexibility**		
Toegel & Nicholson [43]	2005	**Manager multisource ratings**	**Supervisor rating, Subordinate rating**	**Quality of relationship, Gender similarity**
Gyllensten & Palmer [58]	2005	Depression, Anxiety, Stress		
Gyllensten & Palmer [59]	2005	Depression, Anxiety, Stress		
Smither, London, Flautt, Vargas & Kucine [16]	2003	**Manager multisource ratings, Goal specificity**	**Coachee sharing feedback and soliciting ideas for improvement (supervisor**, direct reports, peers)	Goal specificity (MED), Sharing feedback and soliciting ideas (MED)
Luthans & Peterson [15]	2003	**Manager multisource ratings, Manager job satisfaction, Manager organisational commitment, Manager turnover intentions**	**Subordinate job satisfaction, Subordinate organisational commitment, Subordinate turnover intentions**	

*Note*. **Bold = supported**, Normal = not supported, *Italic = Partial support*, MOD = variable explored as a moderator, MED = variable explored as a mediator.

### Individual

#### Self-efficacy

As Table 3 shows, in terms of the individual a number of studies have focused on the changes in the coachee’s self-efficacy, general self-efficacy but also more specific measures such as leader self-efficacy [49], and also goal attainment of the coachee. All the studies investigating a type of self-efficacy found results that support [4, 27, 31, 39, 49, 50] or partially support [40, 51] a positive link between coaching and self-efficacy. Nearly all of the studies investigating the impact of coaching on goal attainment found positive results that support this relationship [31, 47, 52–57], however, one study [41] did not find support for coaching having a positive impact on goal attainment. However, the author did report that the small sample size might have prevented detecting an effect of coaching on goal attainment.

#### Psychological factors

Another area that has received considerable attention in the coaching research is the psychological factors related to the individual including anxiety, stress, depression, resilience and well-being. The studies investigating resilience found positive and significant results for the impact of coaching [31, 52, 53]. Interestingly all three studies had one author in common. The majority of the research examining well-being has been positive [52–54, 57], although Yu et al. [56] found that coaching only resulted in one subscale change in well-being as measured by the scales of psychological well-being and only resulted in a change in positive affect as measured by the Positive Affect Negative Affect Scale. The results for depression have been mixed with some studies finding results that support [31] or partially support [52] the impact of coaching on improving depression whereas others found that this relationship is not supported [57–59]. The studies examining improvements in stress have been more positive than those for depression with most of the studies finding a reduction in stress linked to coaching [45, 53, 57], one found partial support [52] and only a handful found no significant changes [31, 58, 59].

#### Satisfaction and performance

A decent amount of research has investigated the variables discussed above; however, other studies have touched upon satisfaction (work, career and life), performance and self-awareness. The results for these variables have been mixed with some studies finding no support, others partial support and very few finding positive support for coaching leading to improvements in self-awareness, performance or satisfaction. In regards to satisfaction the following studies found positive and significant results: career satisfaction [60], work satisfaction [4] and job satisfaction [15]. However the following studies did not find a link between a satisfaction variable and coaching: workplace satisfaction [31] and life satisfaction [41].

Changes in self-awareness as a result of coaching have been less explored, with only two studies examining it. Both studies used a control group, one found a positive relationship [4] that self-awareness increased through coaching when only examining post-coaching scores, the other study found no significant changes related to self-awareness through coaching when examining post-coaching score while controlling for pre-coaching scores [60]. One study examined self-insight and found that this variable increased significantly between pre- and post-coaching assessments [57].

Job performance has been explored by a handful of studies but the results have not been very positive, one study found a positive and significant link between coaching and objectively measured job performance [61]. However another found partial support for coaching related to a self-reported performance measure [62]. Further, Bozer and Sarros [60] found no significant relationship for those being coached against a control group for self- and supervisor-rated job performance and supervisor-rated task performance.

#### Other outcomes

Other variables related to the individual investigated by researchers which show a positive impact from coaching include: successful and sustained employment for homeless individuals [44], competency change [63], managerial flexibility [64], locus-of-control [39], solution-focused thinking and change readiness [31]. However these outcomes have only been investigated by a single study and their results are far from conclusive.

#### Longitudinal impact

In terms of longitudinal impact of coaching, only a handful of studies have examined the potential effects of coaching after a certain number of months have passed since the intervention. However, the majority of these findings have been positive and support long-term sustained influence of coaching. MacKie [46] examined the transformational leadership scores for their executive coaching group three months after they completed their coaching intervention. Their results showed that although the most significant change in their transformational leadership skills was directly after they completed their coaching intervention there was still a growth in transformational leadership for those individuals no longer being coached.

Finn [40] also examined transformational leadership but only examined the longitudinal impact of coaching on the psychological measures. The data was collected six months after completion of a coaching intervention. Although the first study had a rather small sample size (*n* = 6) the data showed an increase in self-efficacy, developmental support, positive affect, developmental planning and openness to behaviour from pre-coaching to time-3, six months after coaching had ended. However, some of the variables, self-efficacy, developmental support and positive affect, did not show increases between pre-coaching and post-coaching. In the follow up study, self-efficacy and openness to new behaviours increased during the intervention and these levels were maintained but did not increase six months after coaching ended. But developmental planning and support showed decreases after the coaching was completed and there were no significant effects for positive affect. The final study [44] examining the longitudinal effects of a coaching intervention examined the sustained employment of homeless individuals for up to 12 months after they successfully gained employment. They found that compared to the reference group individuals who were supported by a job coach were more likely to sustain employment.

### Organisation

In terms of the organisational outcomes, the most common areas investigated have been transformational leadership, performance as rated by others and manager behaviours, and nearly all of these studies have included multisource ratings. The results for leadership changes as assessed by multiple raters have been mixed. A couple of studies found positive changes due to coaching from all perspectives [15, 16, 43, 46, 65]. However others found positive changes in one type of rater, such as self, but not in all of the other-raters [40, 53, 66]. Two studies found participants of coaching improved in some leadership competences but not all of the competencies [61, 67].

Furthermore, a number of the leadership studies [15, 49, 68] also looked at the impact of coaching on others within the organisation outside of the coachee, such as subordinate’s satisfaction [15, 68], work engagement [68], organisational commitment [15], psychological empowerment [49], psychological strain [68] and turnover intentions [15, 49, 68]–outcomes that are incredibly useful and important for organisations. Two of these studies [15, 49] found data that supported a positive relationship between coaching of a manager and reduced turnover intentions in their subordinates. Additionally coaching was found to increase subordinate’s job satisfaction [15, 68], work engagement [68] and organisational commitment [15]. However, there was no significant effect on psychological empowerment or psychological strain. Interestingly, Biggs et al. [68] and Kochanowski et al.[67] were the only two studies in this review that solely focused on the impact of coaching on the subordinates of a coachee. This method of investigation could be useful for researchers struggling to get access to senior executives undergoing coaching.

One unique coaching evaluation study [54] employed the use of social network analysis to examine the distal or ripple effects of coaching. The authors investigated well-being and quality of interaction changes across both coached and non-coached individuals as well as multirater transformational leadership scores and goal attainment for the coachees. Their results showed that well-being increased for the coached group compared to the non-coached group. Additionally goal attainment and transformational leadership ratings by others were found have increased positively and significantly after the coaching intervention. The results for quality of interaction were not as expected but incredibly informative.

O’Connor and Cavanagh [54] asked participants to rate the frequency and level of positivity and negativity associated with interactions with others at the organisation. From this they were able to analyse the quality of interactions in two directions, out-going and in-going. Essentially, out-going communication was participants’ perceptions of their communications with others and in-going communication was others’ perceptions of communication with participants. When examining differences between the coached and non-coached group they found no change in quality of interactions in either direction in the period between the baseline assessment and the pre-coaching assessment. However, when examining the period between pre- and post-coaching they found differences not only in the perceptions of quality of communication between the coached and non-coached groups but also the direction of communication.

The coached group, on average, perceived the quality of their out-going interactions, as improving and the non-coached group, on average, did not see any improvements in their out-going communication. Where there is some divergence, are others’ perceptions of the quality of the coached and non-coaching groups’ interactions. The in-going communication was significantly more negative for the coached group versus the non-coached group. So the coachees perceived their interactions, as being more positive post-coaching but the people around them did not agree. These findings contrast with the others’ ratings of transformational leadership, which as mentioned above were found to have improved positively. The authors postulate that these results may highlight the potential time lag between a coaching intervention and its positive impact. Also, new patterns or styles of communication initially may be taken negatively especially as the coachee is learning to integrate their newly acquired skills in their day-to-day interactions [54].

In addition, the following outcomes have been examined by a single study: firm growth [39], effectiveness of business improvement plan implementation [69] and meeting behaviours [48]. Although the majority of the results related to these variables were positive and significant as a result of coaching, the limited exploration makes any conclusions impractical.

Remarkably, only six [4, 31, 39, 41, 49, 62] of the 52 studies investigated in this review mentioned ROI, however, none experimentally investigated the ROI of coaching. This may highlight that ROI, although a useful measure for organisations in other areas, may not provide insight related to coaching. However, it could also be possible that it is not definitely clear how coaching impacts coachees and the people around them and therefore with the current literature it is not yet possible to calculate an appropriate ROI value for coaching interventions.

### Mechanisms

The underlying mechanisms were split into three categories: coachee, coach and the relationship. These categories were determined using the active ingredients described by McKenna and Davis [28], however in this current review, any variables related to the coaching methodology, theory, technique or the coaching process was placed in the ‘coach’ category. This is because a coach’s background, experience and training often determine the model or theory employed during their coaching sessions. Although a number of coaches have various techniques at their disposal, limited research has investigated the effectiveness of different coaching techniques within the same experiment, which would allow comparison of the efficacy of different techniques. Additionally within this category, any mediating variables that did not fall under these three categories but do help to explain how coaching improves performance or organisational outcomes were included in the mechanism column in Table 3. For example, Biggs et al. [68] hypothesized that psychosocial work characteristics, such as work-culture support, mediate the relationship between coaching of a manager and improved psychological outcomes of their subordinates.

The following characteristics have been investigated experimentally in the literature: socioeconomic characteristics of the coachee [44], coachee involvement [63], coachee personality [29, 70–72], coachee learning styles [73], coach background [74], working alliance and relationship quality (see Table 3 for exhaustive list), though many of these variables have only yet been explored by one or two studies making it very difficult to make conclusive judgments about how much these factors contribute to the effectiveness of coaching.

#### Coachee

Three studies [42, 44, 48] have investigated coachee demographics and socioeconomic characteristics. Blackman and Moscardo [42] found no significant differences for gender, sector of employment, marital status, whether or not the coachee had children, or length of time in their current position. They did find a significant result for age; finding a greater coaching effective score for individuals aged 50 and over. However when combined with other variables in a regression analysis age was no longer a significant predictor. Further Perkins [48] found that age and verbal IQ level did not have a significant effect on changes in meeting behaviour for executives that underwent a coaching intervention. On the other hand, Hoven et al. [44] who examined the role of coaching for homeless individuals looking for work and sustaining that employment, found that younger individuals (18–24 years) had greater success with a job coach than older individuals as well as those from an Asian background and individuals with a higher education level. This result is all the more striking, as older individuals tend to fare better on the work program investigated in this research. It provides an avenue for targeted coaching interventions that can maximise success of this social program and proves insightful for those interested in applying coaching with younger individuals in organisations.

Another characteristic of coachees that has been examined a handful of times, although under different guises is coachee motivation. Bozer, Sarros and Santora [75] investigated coachee pre-training motivation and found that it was positively related to greater self-reported job performance and self-awareness but had no relationship with supervisor-reported job performance or task performance, or coachee career satisfaction or job affective commitment. Further analysis found the coachee pre-training motivation moderated the relationship between coachee learning goal orientation and self-reported job performance. Bowles et al. [61] found that coachee buy-in was positively linked with growth in leadership competencies but had no impact on participant performance. However, a more recent study, Sonesh et al. [55], found in a group of executive coachees that client motivation was not correlated with the coachee outcome variables of insight and goal attainment. Remarkably, the authors had run the same study with a group of undergraduate students and executive MBA coaches and found a considerably different result. With the student sample, coachee motivation was strongly correlated to the outcome variables and mediation analysis found working alliance to mediate the relationship between coachee motivation and coachee insight. Although this student study was not included in the current review its results are worth highlighting to organisations that have predominantly focused their coaching efforts on executives rather than individuals just entering the workforce.

Furthermore, a group of studies have investigated personality [29, 70–72] of the coachee and the combination of coachee and coach personality but the results from these studies have been mixed and divergent. Jones et al. [70] in a cross-sectional study found that only the facet of Extraversion from the Five Factor Model was correlated with coachee perceived effectiveness of coaching. However, a previous study [72] that utilised the same personality scale but did not examine Extraversion found Conscientiousness positively related to coaching transfer and maintenance. Additionally a positive correlation was found for Openness to Experience and a negative one for Emotional Stability but once a regression analysis was conducted which included general self-efficacy only Conscientiousness was found to make a significant contribution although the model only accounted for 12.6% of the variance of coaching transfer. The other two studies [29, 71] examined the match and mismatch of coachee and coach personality types utilising MBTI. Scoular and Linley [71] found that when coach and coachee differed on temperament, coaching outcome scores were larger, however De Haan et al. [29] failed to replicate these results. They found no contribution of coach or coachee personality, or match or mismatch of personalities.

In terms of other coachee characteristics, the following were found to have a positive and significant link with coaching outcomes: coachee involvement [63], coachee self-efficacy [29], coachee learning styles [73], organisational level of coachee (executive versus middle manager) [61]. Partial support (i.e., the variable or some subscale of the variable was linked with one aspect or variable that was classified as a coaching outcome) was found with the following coachee variables: coachee goal orientation [76], feedback receptivity, learning goal orientation and developmental self-efficacy [75].

#### Coach

In terms of coach characteristics the most widely investigated variable is related to a coach’s behaviours. The results of these studies have been mixed. One found a positive and significant result for coach’s facilitative behaviour [49] and improvements in leader role-efficacy and leader’s trust in subordinates. Furthermore, a single study [47] investigating the impact of coach affiliation and dominance behaviour during the first coaching session found only dominance behaviour to be predictive of client goal attainment at the end of the coaching intervention. Additionally, similarity of coach and client on interpersonal dominance and affiliation was also predictive of goal attainment.

Another study found partially supportive results for a coach’s role [39] which positively impacted client’s self-efficacy, which in turn lead to firm growth. Furthermore De Haan et al. [73] found that a number of coaching behaviours positively predicted client’s perceived helpfulness of coaching but no specific behaviour was significant. In a follow up study, De Haan et al. [29], found that a client’s perception of the range of techniques a coach possesses is predictive of a client’s perception of the effectiveness of coaching. On the other hand, however, Sonesh et al. [55] found that none of the coaching behaviours they investigated, including rapport building, coaching authentically, providing content, boundary setting and regulating motivation, were positively associated with goal attainment or coachee insight.

The next area examined within coach characteristics relates to inherent qualities of a coach. For example, Gatling and Harrah [77] found that a coach’s authentic leadership predicted coach’s perceived effectiveness of coaching on their clients. Two other studies, however, found no relationship between coach’s psychological mindedness [55] and coach’s features [42] when predicting coaching outcomes. However, Blackman and Moscardo [42] did find that the similarity of coach to coachee did predict effectiveness of coaching.

The only other variables investigated related to coach attributes that influence the outcomes of coaching fall under coach training and techniques. The studies in this area although sparse have been relatively more positive than other aspects of the coaches. For instance, MacKie [46] found that the amount of adherence to the strength-based methodology was a significant predictor of client’s change in transformational leadership behavior, with greater adherence resulting in a greater positive leadership change. Furthermore, Bozer et al. [74] investigated the effects of coaches’ academic background in psychology and credibility on coaching effectiveness. Their results showed that a coach’s academic background in psychology positively influenced greater coachee self-awareness and supervisor-rated job performance of coachees. However, it had no significant effects related to self-reported job performance, career satisfaction or job affective commitment. Additionally, coach credibility was found to positively relate to self-reported job performance.

#### Relationship

As the last two sections clearly show, the majority of coach and coachee characteristic variables have only been explored by a single study. However, one characteristic that been explored in rather more depth would be the relationship between the client and coach, which has been examined a number of times over the last decade.

One measure of the relationship between coach and coachee that appears prevalently in the literature is working alliance, a concept that coaching has adopted from the psychotherapy space. Working alliance has not only been investigated as a predictor for coaching effectiveness but has been examined as a mediator between coachee and coach inputs and coaching outcomes. In one such investigation [27, 78] working alliance was found to be positively related to changes in self-efficacy for a group of coached executives and working alliance was found to mediate the relationship between number of coaching sessions and coachee self-efficacy. Further analysis found the following significant correlates of working alliance: coachee motivation to transfer, coachee perception of supervisor support, coach's self-efficacy with regard to facilitating learning & results, and number of coaching sessions, however coach relational skills and coach communication skills were surprisingly not significant. In addition, the authors examined how discrepancy between coachee and coach’s perceptions of working alliance would impact self-efficacy development. Their results showed that coachees of coaches that underestimated their working alliance had greater growth in self-efficacy compared to those coaches that were accurate or overestimated their working alliance.

In an other study examining working alliance, De Haan and colleagues [29] investigated the relationship between working alliance and client’s perceived coaching effectiveness and they found that the client’s perception of the working alliance but not the coach’s perception of the strength of the relation was positively correlated with the client’s perceptions of coaching effectiveness. De Haan et al. [29] also investigated working alliance as a mediator of certain coachee and coach inputs and coaching outcome. They used client self-efficacy as a client input rather than outcome of coaching. Nevertheless, they found that working alliance fully mediated the relationship between client self-efficacy and client’s perceived effectiveness of coaching. Working alliance also partially mediated the relationship between a client’s perception of their coach’s range of techniques and client’s perceptions of the effectiveness of coaching. Their non-significant results related to personality prevented this from being examined in such a mediation analysis.

However, not all of the studies examining working alliance have found significant effects. Sonesh et al. [55] found that in their executive coaching sample working alliance was correlated with coachee insight but had no relationship with goal attainment. Also mediation analysis found that working alliance did not mediate the relationship between coach psychological mindedness, coach behaviours and coachee motivation and the outcomes of coaching: coachee insight and coachee goal attainment.

Besides working alliance, other studies have investigated other concepts that capture the quality of the coach-coachee relationship. For instance, Gan and Chong [79] investigated the components of a coach-coachee relationship by examining rapport, trust, commitment and coach-coachee match related to coaching effectiveness. Through regression analysis they found only rapport and commitment to be related to coaching effectiveness. Although coach-coachee match in this study was not significant other studies have found coach-coachee similarity, as well as gender similarity, to be related to greater coaching effectiveness as well as the quality of the relationship between coachee and coach [42, 43, 80].

In an attempt to further understand what components underlie an effective coaching relationship Grant [31] examined autonomy support, satisfaction with coach-coachee relationship, the extent to which the relationship was similar to an “ideal” coaching relationship and goal-focused coaching relationship. All of the variables outside of satisfaction with the coach-coachee relationship showed positive and significant correlations with goal attainment. However, once controlling for goal-focused relationship the other components became non-significant. Additionally, there were no significant correlations between any of the different aspects of the coach-coachee relationship and any of the individual outcomes examined in the study, including self-insight, psychological well-being, anxiety, stress and depression.

### Implications

As discussed above and shown in Table 3, there is considerable variation among the coaching outcomes and coaching mechanisms explored in the studies examined in this review. Unfortunately, it is clear there is not enough data to make a definitive judgment about the effectiveness of coaching on each of the outcomes investigated in these studies because few of them have been investigated multiple times, with experimental rigour or with large enough sample sizes. Additionally, some of the papers in this review did not report an effect size if the relationship between outcomes variables and the coaching intervention were found to be non-significant, which can potentially bias any conclusions that could be made. However, the results above do lean towards coaching being an effective intervention that helps individuals in terms of their self-efficacy, goal attainment and organisations in terms of their leadership but it also benefits organisations indirectly through the individual.

For instance, coaching has been found to impact an individual’s self-efficacy and a considerable amount of research has investigated self-efficacy and performance in the work place. Drawing on a broad range of research and not specifically coaching studies a meta-analysis of over 100 studies found that self-efficacy explained 28% of the variance in work place performance, which is considered to be a large effect size [81]. Furthermore, as well as improvements in psychological factors, such as well-being and resilience, being of great benefit to the individual they are also beneficial to the organisation through improved work performance (e.g., well-being and work performance: Robertson, Birch and Cooper [82] and Wright and Cropanzano [83]) and greater resilience has been linked to desirable employee attitudes, behaviours and performance [84].

Alongside self-efficacy, goal attainment has been explored as an outcome of coaching interventions. Higher or continued goal attainment leads to greater satisfaction on an individual level. Additionally, countless research has also shown the setting of specific goals of certain levels of difficulty have been linked to increased productivity, performance and even organisational profitability [85]. This showcases that although the majority of coaching research to date has focused on the benefits of coaching to the individual these benefits do also extend to the team and organisational level.

Investigation of other variables such as satisfaction and job performance, which are key work outcomes, have not found definitive support for coaching increasing these outcomes but this may be due to the inherent nature of the intervention. Due to the customised nature of coaching, it is hard to generalise different aspects of performance that are likely to change with an individual. For example, two sales people may both undergo coaching, one may be looking to improve their relationships with clients and the other looking to improve their time management skills. They both may see a considerable improvement in these areas but this may not be accurately reflected in a standardised measure of performance, which is a requirement for an academically rigorous study. Furthermore, it may take longer than the duration of these experimental procedures for the positive effects of coaching to manifest themselves in self-performance ratings as well as ratings from others, such as supervisors and subordinates. In addition, work or career satisfaction measures could also be misleading as a coaching intervention may result in some individuals deciding they would like to leave their job or organisation which is likely to decrease satisfaction but is beneficial to the individual and organisation in the long-term.

In terms of direct impact on organisations the distal outcomes of the research show coaching helps to improve leadership and manager behaviours as well as improving ratings given by individuals in close proximity to the coachee. Furthermore coaching has been shown to impact subordinates in a meaningful way through reduced turnover intentions and increased satisfaction and organisational commitment. Although it is difficult to assign a ROI value to this impact and researchers have all but relinquished futile attempts to do so, this review does show that coaching interventions can have far reaching impact on organisations. However, more research investigating distal outcomes, such as changes in ratings from supervisors, peers and subordinates, and organisational specific outcomes such as productivity, revenue generations, sales commissions, etc., is needed to cement the advantages and use of coaching in organisations.

Although not the direct focus of this review a number of studies employed coaching interventions in tandem with training [62] or workshops [64, 66, 67] or embedded within leadership development programs [40, 52, 68]. The majority of these studies found positive effects of coaching within this format. Although this design is not effective for isolating the unique impact of coaching it does more genuinely reflect the practical application of coaching in an organisational setting.

## Discussion

The main aim of this review was to examine the research pertaining to the effectiveness of coaching in an organisational setting in the hopes of providing practical insight to the users and buyers of coaching. A number of positive associations have been highlighted above but the research is still relatively nascent and there are a number of gaps and issues that need to be addressed. For instance, in some studies self-efficacy is used as an outcome variable for measuring the effectiveness of coaching, for example in the Baron and Morin [27] discussed above and also in one leadership study [49]. The authors in the leadership study, after conducting a focus group, used leader efficacy as one of the outcome criteria for evaluating the effectiveness of the coaching intervention. However, in other studies, self-efficacy, as an individual difference or trait, has been investigated as a predictor of the effectiveness of coaching. In one such cross-sectional study, De Haan et al. [29] measured the general self-efficacy of the coachee (self-report) and found that this correlated highly (*r* = .25 with *p* < .01) with the client’s perceived effectiveness of coaching. This highlights a great concern within the current coaching research; there is no agreed or definitive list of outcomes of coaching. Consequently, it is unclear whether self-efficacy predicts coaching effectiveness, is a result of coaching or whether there is a reciprocal relationship between self-efficacy and coaching.

Self-efficacy is not the only variable with which there is an issue. It is clear from this review that there is no consistency in terms of outcome measures. Many of the outcome measures that have been examined have been leveraged from other domains, such as psychotherapy, learning and training, which is understandable in terms of exploration of coaching as an intervention. However, for coaching to progress to an advanced level in terms of academic investigation, the research community needs to assess the suitability of these outcome measures for coaching. If these outcome variables are found to be unsuitable for measuring the effectiveness of coaching in terms of encompassing all the benefits of coaching accurately, then new outcome measures must be devised specifically for coaching.

Additionally, very few studies have examined the interaction between variables that underlie the mechanisms of effective coaching, notable exceptions would be studies by Baron and Morin [27], De Haan et al. [29] and Sonesh et al. [55]. In these studies the authors investigated working alliance as a mediator and in one, a potential moderator between coaching effectiveness and coaching characteristics coachee inputs, and coach inputs. Furthermore, they attempted to investigate what might predict working alliance by examining its correlates. Although these are only a limited number of studies the results are an initial small step in the right direction to better understand how coachee, coach and relationship variables potentially interact with one another to influence the effectiveness of coaching.

### Issues with the research

As mentioned earlier, coaching is a unique intervention customised to the coachee and often there is limited comparability across coachees for a number of factors, such as goals, background and contextual factors. These nuances of coaching make it very difficult to measure the effectiveness of coaching in an academically rigorous study. Furthermore, it makes it difficult to compare coaching research to the research done to measure the effectiveness of other interventions such as classroom-training though often coaching falls into the same bucket as these interventions. Although coaching is a tough intervention to measure it does not open the door to less stringent and conscientious research. Furthermore, the majority of the issues faced by coaching researchers are not isolated to the coaching arena but have been faced by other domains within the organisational, management and psychological research literature. During this review a number of concerns related to the coaching research have been found.

There appears to be somewhat of an inflation of studies examining the effectiveness of coaching where single data sets have been split across multiple papers, sometimes without clearly stating that the effectiveness findings have already been published elsewhere. This can lead to false interpretation by individuals who are looking to better understand the effectiveness of coaching but do not have an academic background. Because of the difficulty associated with collecting coaching effectiveness data it is understandable to collect as much information as possible within statistically sound limits. It is also appropriate to split investigation of different aspects across papers however repeatedly including coaching effectiveness results that have already been previously published without stating so can be incredibly misleading. Further, other studies have potentially utilised different analysis techniques on data sets that have already been published with alternative analysis with possibly different results. Also there have been some inaccuracies when reporting previous findings—previous correlation data has been stated as causation and insignificant findings, which don’t show the effectiveness of coaching have been ignored when summarising previous research.

Furthermore, there are methodological issues in the coaching literature that need to be addressed. There is an overreliance on self-report measures and retrospective data, very small sample sizes and incredibly limited use of objective outcome measures or measurement of distal and longitudinal impact of coaching. The use of self-report measures and cross-sectional design is a common problem across the organisational and management literature. Self-report measures are useful when assessing an individual’s perceptions or satisfaction with an intervention but they are not appropriate when assessing objective organisational outcomes. For example, if an organisation has invested a considerable sum of money to provide coaching to individuals, those individuals are potentially going to be prone to answering questions related to their job performance in an *organisationally desirable* manner. Furthermore self-report data used to predict correlations on other self-report data can be misleading as the correlations could be a result of the mechanism of assessment rather than an actual relationship between the variables. The use of this type of data collection can introduce a number of biases that can influence the results of a study [23, 86].

Self-report measures and cross-sectional designs are often used within organisational research because it is frequently difficult for academics to get access to individuals in organisations. Furthermore when access is granted it is often limited especially as the seniority of individuals increases. These problems have been over come in other areas of organisational research and it is important that coaching learns from these other domains. For example, all measurement methods whether self-report or not have associated measurement error. The amount of measurement error varies with the method of measurement but structural equation modelling (SEM) has been widely used to estimate and account for measurement error/bias in academic studies. However, only one study [39] included in this review utilised SEM [22, 23, 86].

Additionally, the psychological literature has more recently been plagued by concerns around the replicability of psychology studies. These concerns should also be considered by academics investigating coaching phenomena. A recent project to reproduce the results from a 100 published psychology studies found that less than half of the results were reproduced even when using available original materials. They did find that the success of replication appeared to be related to the strength of the original finding [87]. The results from this project should implore coaching researchers to make a concentrated effort to reproduce the results from earlier findings. Inevitability, research can only build and learn from what has already been done but it is important to ensure that previous findings are accurate.

It is important to state that coaching research has improved a lot, even over just the last 5 years; more viable studies are being published every year and a number of great experimental studies (such as Grant [57], Ladegard and Gjerde [49] and MacKie [46]) have been published over the last 2 years and this momentum needs to continue and actually increase somewhat. Whilst the requirements of research rigor are not those of practitioners, the only way to really investigate many of these issues is the randomised control study with all the costs and difficulties associated with that. What however is always most difficult to measure is the outcome over time. Most studies have accepted coachee self-reported “improvement” immediately after the ending of the coaching assignment. A better indicator would be reports from others at work as well as behaviour changes immediately after the coaching but also six months and a year later. Although not a randomised control study, a couple of the studies in this review [40, 46] utilised wait-listed participants as a control group. This is practical for organisational settings because these individuals still do receive coaching in the next round and it is an effective mechanism for creating a control group that is exposed to the same organisational environment as the coachees. This experimental design also allows for a longitudinal assessment of the first coached group.

As an industry, coaching needs more stringent methodology, statistical analysis and larger sample sizes to increase the generalizability of the coaching effectiveness findings. Furthermore, more objective and multisource ratings of outcomes are needed. Turnover of raters can be an issue in organisational settings and this potentially discourages researchers from using such measures [17] but a recent study by MacKie [46] demonstrated that a moderate turnover in raters did not adversely impact the reliability of the ratings. This confirms the validity of utilising multirater methodology coaching effectiveness research in organisational settings. Additionally, we need more coaching studies specifically addressing mechanisms underlying the impact of coaching. Finally, research needs to explore the moderators and mediators of effectiveness because when this happens, it is a sign of the maturity of a subject matter.

In light of the issues related to the coaching effectiveness research discussed in this section it seemed pertinent to categorise the studies included in this review based on the potential risk of bias that may be associated with the study design and methodology. Table 4 provides an overview of the sources of potential bias that may be associated with each study, any mechanisms that may have been employed to overcome or reduce these biases and a rating of the risk of bias that is attributable to each study. Following on from this, any findings from studies with a low risk of bias have been summarised in Table 5. Table 5 summarises the results from these 12 low risk studies that have been found to be significant (supported), partially significant (partially supported) and not significant (not supported). As these findings are a result of good research design and methodology, it would be sensible to use these findings as a foundation for future coaching effectiveness research. The weight attributed to these findings in Table 5 should be considered to be greater than that of the findings from the medium and high risk of bias studies. However, when examining Table 5 it is clear that very few findings, either individual-level outcomes or organisational-level outcomes, have been replicated in these studies, which limits their generalizability.

**Table 4:** Overview of risk of biases for each individual study.

Author	Year	Sources of potential biases	Potential biases	Mechanisms to counter biases	Rating of risk of bias
Gan & Chong [79]	2015	Cross-sectional design	Common method variance	None employed	High risk
		Self-report	Sampling bias		
		57% response rate—no differential analysis of dropouts	Social desirability bias		
			Attrition bias		
Sonesh, Coultas, Marlow, Lacerenza, Reyes & Salas [55]	2015	Cross-sectional design	Common method variance	Self-report data from coach and coachee	High risk
		Self-report	Sampling bias		
			Social desirability bias		
Biggs, Brough & Barbour [68]	2014	Nonrandomised group allocation—pre-existing differences between groups	Selection bias	Control group	Low risk
		No differential analysis of dropouts	Attrition bias	Other-raters	
				Pre & post assessment	
Hoven, Ford, Willmot, Hagan & Siegrist [44]	2014	Nonrandomised group allocation	Selection bias	Control group	Low risk
				Objective outcome	
				Longitudinal outcome	
Ladegard & Gjerde [49]	2014	Small sample size	Selection bias	Control Group	Low risk
		Nonrandomised group allocation	Attrition bias	Other-raters	
		73% response rate—no differential analysis of dropouts		Pre & post assessment	
Jones, Woods & Hutchinson [70]	2014	Small sample size	Common method variance	None employed	High risk
		Cross-sectional design	Social desirability bias		
		Self-report	Sampling bias		
Gatling & Harrah [77]	2014	Cross-sectional design	Common method variance	None employed	High risk
		Self-report	Social desirability bias		
			Sampling bias		
Grant [57]	2014	Small sample size	Common method variance	Pre & post assessment	Medium risk
		Self-report	Social desirability bias		
			Sampling bias		
Mackie [46]	2014	Small sample size	Selection bias	Control group	Low risk
		Nonrandomised group allocation		Multiple raters	
				Pre & post assessment	
				Longitudinal assessment	
				ANCOVA	
				Analysis of rater consistency	
O’Connor & Cavanagh [54]	2013	Small sample size	Selection bias	Control group	Low risk
				Multiple raters	
				Pre & post assessment	
Ianiro, Schermuly & Kauffeld [47]	2013	Small sample size	Sampling bias	Other-raters	Medium risk
		Self-report coaching success	Social desirability bias	Pre & post assessment	
Smith & Brummel [63]	2013	Small sample size	Common method variance	Experts coded responses	High risk
		Cross-sectional design	Sampling bias		
		Self-report	Social desirability bias		
De Haan, Duckworth, Birch & Jones [29]	2013	Cross-sectional design	Common method variance	Self-report data from coach and coachee	High risk
		Self-report	Sampling bias		
			Social desirability bias		
Grant [31]	2013	Small sample size	Common method variance	Pre and post assessment	Medium risk
		Self-report	Sampling bias		
		No differential analysis of dropouts	Social desirability bias		
			Attrition bias		
Nieminen, Smerek, Kotrba & Denison [66]	2013	Nonrandomised group allocation—pre-existing differences between groups	Selection bias	Control group	Low risk
				Multiple raters	
				Pre and post assessment	
Bozer & Sarros [60]; Bozer, Sarros & Santora[75]; Bozer, Sarros & Santora [74]; Bozer, Joo & Santora [80]	2012; 2013; 2014; 2015	Nonrandomised group allocation	Selection bias	Control group	Low risk
				Supervisor ratings	
				Pre and post assessment	
Vidal-Salazar, Ferrón-Vilchez and Cordón-Pozo [69]	2012	Small sample size	Selection bias	Control group	Medium risk
		Cross-sectional design		Other-raters	
		Nonrandomised group allocation			
Bright & Crockett [62]	2012	Pre-existing differences between groups	Selection bias	Control group	Medium risk
		Self-report	Social desirability bias	Randomised group allocation	
		New measure utilised—no validity or reliability information given	Measurement bias	Pre and post assessment	
Blackman & Moscardo [42]	2012	Cross-sectional design	Common method variance	None employed	High risk
		Self-report	Social desirability bias		
			Sampling bias		
Crompton [39]	2012	Cross-sectional design	Common method variance	Control group	Medium risk
		Self-report	Social desirability bias	Objective outcome measure	
			Sampling bias	Structural Equation Modelling	
Ladegård [45]	2011	Self-report	Social desirability bias	Pre & post assessment	Medium risk
		50% response rate	Attrition bias	Longitudinal assessment	
		New measure utilised—no validity or reliability information given	Measurement bias	Differential analysis of dropouts	
De Haan, Culpin & Curd [73]	2011	Small sample size	Common method variance	Pre and post assessment	Medium risk
		Self-report	Sampling bias		
		28% response rate—no differential analysis of dropouts	Social desirability bias		
			Attrition bias		
Scriffignano [76]	2011	Cross-sectional design	Common method variance	None employed	High risk
		Self-report	Social desirability bias		
		67% response rate—no differential analysis of dropouts	Sampling bias		
			Attrition bias		
Richardson [41]	2010	Small sample size	Selection bias	Control group	Low risk
		Nonrandomised group allocation		Other-raters	
				Pre and post assessment	
Kochanowski, Seifert & Yukl [67]	2010	Small sample size	Selection bias	Control group	Low risk
		No differential analysis of dropouts	Attrition bias	Other-raters	
				Pre and post assessment	
Leonard-Cross [4]	2010	Cross-sectional design	Common method variance	Control group	High risk
		Self-report	Sampling bias		
		Nonrandomised group allocation	Social desirability bias		
Cerni, Curtis & Colmar [65]	2010	Small sample size	Selection bias	Control group	Low risk
		Nonrandomised group allocation		Multiple raters	
				Pre and post assessment	
Grant, Green & Rynsaardt [53]	2010	Small sample size	Selection bias	Control group*	Medium risk
		Self-report*	Attrition bias	Multiple raters*	
		Multirater feedback provided to only coached group	Confounding bias	Pre and post assessment	
		No differential analysis of dropouts			
Grant, Curtayne & Burton [52]	2009	Small sample size	Common method variance	Control group	Medium risk
		Self-report	Social desirability bias	Pre and post assessment	
		No differential analysis of dropouts	Selection bias		
			Attrition bias		
Baron & Morin [27]; Baron, Morin & Morin [78]	2009; 2011	Self-report for outcome measure	Common method variance	Self-report data from coach and coachee	Medium risk
		58% response rate—no differential analysis of dropouts	Social desirability bias	Pre and post assessment	
			Selection bias		
			Attrition bias		
Perkins [48]	2009	Small sample size	Sampling bias	Coach/author coded meeting behaviours	Medium risk
		New measure utilised—some validity and reliability information given	Measurement bias	Pre and post assessment	
Moen & Skaalvik [97]; Moen & Federici [98]; Moen & Federici [50]	2009; 2012; 2012	Small sample size	Common method variance	Control group	Medium risk
		Self-report	Social desirability bias	Randomised group allocation	
		New measures utilised—no validity or reliability information given	Selection bias	Pre and post assessment	
			Measurement bias		
Stewart, Palmer, Wilkin & Kerrin [72]	2008	Cross-sectional design	Common method variance	None employed	High risk
		Self-report	Social desirability bias		
		New measures utilised—some validity or reliability information given	Sampling bias		
			Measurement bias		
Yu, Collins, Cavanagh, White & Fairbrother [56]	2008	Small sample size	Common method variance	Pre and post assessment	Medium risk
		Self-report	Social desirability bias		
		No differential analysis of dropouts	Sampling bias		
			Attrition bias		
Finn (Study 1) [40]	2007	Small sample size	Common method variance	Control group	Medium risk
		Self-report	Social desirability bias	Random group allocation	
		New measures utilised—some validity or reliability information given	Selection bias	Pre and post assessment	
			Attrition bias	Longitudinal assessment	
				Differential analysis of dropouts	
Finn (Study 2) [40]	2007	Small sample size	Selection bias	Control group	Low risk
		New measures utilised—some validity or reliability information given	Attrition bias	Random group allocation	
				Multiple raters	
				Pre and post assessment	
				Longitudinal assessment	
				Differential analysis of dropouts	
Bowles, Cunningham, De La Rosa & Picano [61]	2007	Small sample size	Sampling bias	Other-raters	Medium risk
				Pre and post assessment	
Orenstein [99]	2006	Small sample size	Sampling bias	Other-raters	High risk
		Cross-sectional design	Measurement bias		
		New measure utilised—no validity or reliability information given			
Evers, Brouwers & Tomic [51]	2006	Small sample size	Common method variance	Control group	Medium risk
		Self-report	Social desirability bias	Pre and post assessment	
		New measure utilised—no validity or reliability information given	Sampling bias		
		Pre-existing differences between groups	Measurement bias		
Scoular & Linely [71]	2006	Cross-sectional design	Common method variance	Self-report data from coach and coachee	High risk
		Self-report	Social desirability bias		
			Sampling bias		
Jones, Rafferty & Griffin [64]	2006	Small sample size	Common method variance	Control group	Medium risk
		Self-report	Social desirability bias	Pre and post assessment	
		Several participants attend a course during the intervention	Sampling bias		
			Confounding bias		
Toegel & Nicholson [43]	2005	Self-report	Common method variance	Multiple raters	Medium risk
		60% response rate—no differential analysis of dropouts	Social desirability bias	Pre and post assessment	
			Sampling bias		
			Attrition bias		
Gyllensten & Palmer [58]	2005	Small sample size	Common method variance	Control group	Medium risk
		Self-report	Social desirability bias	Pre and post assessment	
		Nonrandomised group allocation	Sampling bias		
Gyllensten & Palmer [59]	2005	Cross-sectional design	Common method variance	Control group	High risk
		Self-report	Social desirability bias		
		Nonrandomised group allocation	Sampling bias		
Smither, London, Flautt, Vargas & Kucine [16]	2003	Nonrandomised group allocation	Selection bias	Control group	Low risk
				Multiple raters	
				Pre and post assessment	
Luthans & Peterson [15]	2003	Small sample size	Sampling bias	Multiple raters	Medium risk
				Pre and post assessment	

Note:

*not used for all outcome measures.

**Table 5:** Variables investigated by low risk studies.

Author	Year	Supported	Partial support	Not supported
Biggs, Brough & Barbour [68]	2014	**Subordinate work engagement, Subordinate job satisfaction, Subordinate perception of strategic alignment (MED), Subordinate perception of work-culture support (MED)**		Subordinate turnover intentions, Subordinate psychological strain, Subordinate perception of job demands (MED) Subordinate perception of supportive leadership (MED)
Hoven, Ford, Willmot, Hagan & Siegrist [44]	2014	**Successful employment, Sustained employment, Age (MOD),Ethnicity (MOD), Educational level (MOD)**		
Ladegard & Gjerde [49]	2014	**Leader efficacy, Leader's trust in subordinates, Turnover intention (subordinates), Coach facilitative behaviour (Mechanism)**		Psychological empowerment (subordinates)
Mackie [46]	2014	**Transformational leadership, Strength-based methodology (Mechanism)**		
O’Connor & Cavanagh [54]	2013	**Well-being, Goal attainment, Transformational leadership, Quality of communication (self), Quality of communication (others) [NEGATIVE]**	*Well-being of those close to coachee*	Quality of communication across organisation
Nieminen, Smerek, Kotrba & Denison [66]	2013		*Leadership behaviours (self)*	Leadership behaviours (subordinates, peers & supervisors)
Bozer & Sarros [60]; Bozer, Sarros & Santora [75]; Bozer, Sarros & Santora [74]; Bozer, Joo & Santora [80]	2012; 2013; 2014; 2015	**Career satisfaction**	*Coachee pre-training motivation (MOD*, *Coachee feedback receptivity (MOD)*, *Coachee learning goal orientation (Mechanism)*, *Coachee developmental self-efficacy (Mechanism)*, *Coach’s academic background in psychology (Mechanism)*, *Coach’s credibility (Mechanism)*, *Gender similarity (Mechanism)*, *Perceived similarity (Mechanism)*	Self-awareness, Job affective commitment, Job performance (self & supervisor), Task performance (supervisor)
Richardson [41]	2010	**Working alliance**	*Coachee goal orientation (Mechanism)*	Goal attainment, Life satisfaction
Kochanowski, Seifert & Yukl [67]	2010		*Influence of behaviour (subordinates)*	
Cerni, Curtis & Colmar [65]	2010	**Multifactor leadership questionnaire (subordinates)**		
Finn (Study 2) [40]	2007	**Transformation Leadership (peer rating)**	*Self-efficacy*, *Developmental support*, *Positive affect*, *Openness to new behaviours*, *Developmental planning*	Transformation Leadership (self & supervisor rating)
Smither, London, Flautt, Vargas & Kucine [16]	2003	**Manager multisource ratings, Goal specificity, Coachee sharing feedback & soliciting ideas for improvement (supervisor)**		Coachee sharing feedback and soliciting ideas for improvement (direct reports, peers), Goal specificity (MED), Sharing feedback and soliciting ideas (MED)

*Note*. **Bold = supported**, Normal = not supported, *Italic = Partial support*, MOD = variable explored as a moderator, MED = variable explored as a mediator.

Nevertheless, practitioners and organisations can leverage the findings in Table 5 to highlight areas in which stringent academic research has found links between a coaching intervention and individual outcomes, such as well-being, goal attainment, career satisfaction, and organisational outcomes, such as subordinate work engagement and job satisfaction and other-ratings of leadership behaviours. However, as mentioned it is also important to highlight that in order for these relationships to be definitive, it is imperative that future coaching research replicates the studies and findings from Table 5.

### Limitations and Future Research

#### Quantitative versus qualitative

This review focused on coaching papers that incorporated some sort of quantitative analysis in their results and did not include papers that were purely qualitative. This is by no means a reflection on the usefulness of qualitative versus quantitative analysis but rather a mechanism by which to segment the coaching research. Qualitative analysis provides a unique insight into complex phenomenon [88], such as coaching, and can be incredibly useful to guide where quantitative research should be focused. As such we recommend that researchers continue to leverage qualitative research but not to the extent that they forgo quantitative analysis. Qualitative research is more likely to be used during the initial stages of exploration of a subject matter [10] and coaching is now at a point where more randomised control studies are needed as well as meta-analyses that include more than 25 studies. Additionally coaching is not the only intervention that has struggled with amassing adequate research investigation. Mentoring has also run into similar problems in the academic domain. However, researchers in this domain have combined laboratory and field settings to further understand the concepts underlying mentoring relationships [89]. Sonesh at al. [55] have attempted this combination of lab and field settings but unfortunately their results were opposing, potentially highlighting whether participant type, student or executive, may moderate either the effectiveness or the mechanism via which coaching is significant.

#### Correlation studies

This review did include papers that used cross-sectional or retrospective design, as well as those with more experimental rigour, it is impossible to determine causation from such studies however with few studies meeting more stringent requirements it was necessary to include such papers. Within some of these retrospective studies, participants were assessed after the coaching but no assessment was carried out before. Although these studies did support coaching as an effective intervention, these conclusions are based on poor methodological design. A number of things can potentially influence their judgment and lead to inaccurate conclusions, such as hindsight bias, where memories could potentially influence the responses of participants [51]. Furthermore, individuals who undergo coaching could be inclined to report that the coaching was successful as it may serve them best. Research has shown that a considerable portion of executive coaches are hired to address derailing behavior so in this instant, for example, a coachee may be more likely to respond that coaching has worked [25]. Cognitive dissonance [90] could also be a factor as coaching is a very expensive intervention and coachees have to dedicate time and effort to the coaching process. Consequently, to avoid cognitive dissonance they may be more likely to report more favorable outcomes in relation to the intervention. Further, in some evaluation studies the research or evaluation and in some cases the outcome variable was reported by the coach providing the coaching. Although we do not doubt that these coaches endeavored to be neutral whilst collecting their data, it does work in the self-interest of the coach to report results that favour their practice, which can potentially tarnish the usefulness of their results [17].

Although these less methodologically stringent papers have issues, they are helpful in understanding where future studies should focus and areas that may prove fruitful in determining mechanisms or models for coaching effectiveness. However, a stringent level of academic rigour must be maintained in future coaching research. Clearly such research is not easy to do in organisational settings and due to the inherently customised nature of the intervention. It is also difficult to get organisations to participate and can be incredibly time consuming for participants. However, experimentally sound quantitative studies are needed to determine causation between the fundamental variables of coaching [91].

#### Coaching in tandem with other development interventions

This review included those studies that incorporated a coaching intervention in tandem with multisource feedback, training and leadership development, which makes it difficult to isolate the specific effects of coaching. However, these studies are more closely related, in terms of contextual and environmental factors, to organisational settings where coaching is very rarely used in isolation [17]. Additionally, this review did include studies that utilised either an internal (non-supervisory) coach or external coach. Recent research [21] has found that multisource feedback combined with coaching is not as effective as coaching on it is own. Additionally, it was found that internal coaches were more effective than external coaches. However, as mentioned above, the analysis was exploratory and cannot rule out confounded effects. Therefore it remains unclear whether these factors are genuine moderators that influence the effectiveness of coaching and as such this is something that needs to be explored specifically with future research.

#### Lack of longitudinal research

In terms of understanding the long-term impact of coaching, very few studies examined any sort of longitudinal influence of coaching even though the continued effectiveness of coaching interventions is an incredibly important area for organisations to understand. The influence of coaching a number of months or years after a coaching intervention can prove insightful in terms of cost-benefit analysis and also potentially determine the suitability of the intervention in a particular scenario. Although the handful of longitudinal studies in this review found some support for the longitudinal impact of coaching, these results are far from conclusive and highlight the need for longitudinal investigation of coaching in organisations. Additionally researchers need to ground any longitudinal research by researching time lags and models associated with the uptake of coaching and also avoid too frequent assessment, which may exacerbate drop out or biases [68].

#### Overreliance on self-report

A considerable number of the studies included in this review relied on self-reports of outcome measures. Previous research has show that there can be inconsistency between self-reports and other-reports (e.g., by the supervisor or coach) when evaluating change in the coachee: self-reports tend to overestimate the effects of coaching interventions. Self-report evaluations are important in coaching, largely due to the customised nature of the intervention, as they enable researchers to understand the impact of coaching on performance for that individual. However, self-report data does not capture the ripple effect of coaching through out an organisation. To capture the more distal influence of coaching in an organisational setting, studies need to include other-ratings, ideally multisource ratings but also objective organisational measures [54].

#### Gaps in the research

A number of areas and questions are yet to be explored in coaching. For instance, are there cultural differences with the effectiveness of coaching or do different mechanisms explain how coaching is effective in different cultures. Although some research as examined coaching effectiveness in other cultures (e.g., Gan & Chong [79]) the majority of coaching research has focused on western countries and cultures. Additionally, group differences such as gender or age as yet have not been explored in any depth in the space of coaching. Organisations tend to reserve coaching for senior individuals due to the intrinsic cost of coaching. However, some studies in this review have highlighted that coaching may be more effective in younger individuals just entering their careers [44, 55]. Furthermore, the dark side of personality has yet to be explored as a moderator of coaching effectiveness even though initial work has examined the bright side of personality.

#### Neuroimaging techniques and neuroscience in coaching

Moreover, coaching research should widen its horizons in terms of alternative mediums for data collection, such as videoing coaching sessions or observing behaviours in situ but it should also leverage neuroimaging techniques. It is important to state here that there is some controversy surrounding neuroscience in coaching. Unfortunately, a number of individuals have coined phrases such as neuroleadership and neurocoaching and use them as a marketing tool without any bases or understanding of neuroscience. The following discussion of neuroimaging and neuroscience is in no way related to this movement but is a call for academic research that examines neuroscience within a coaching context.

A number of neuroimaging studies have investigated the impact of cognitive behavioural therapy and other interventions on mental health issues (e.g., Porto, Oliveira, Mari, Volchan, Figueira and Ventura [92]) but none have examined the impact of coaching on the brain and very few have examined the impact of talking therapies on healthy individuals. However, these types of studies can potentially provide some insight into coaching techniques in terms of what mechanism is leading to a positive effect, for instance, considering non-judgmental attention. In the majority of situations, judgment equates to criticism; judgment is an evaluation of what has been said, it may be a good or bad evaluation but it is essentially conveying information about the value of what the client has said and this could potentially undermine the client’s thought process. Neuroimaging studies have investigated the impact of criticism on the functional connectivity in an individual’s brain. One such study [93], utilising fMRI (functional magnetic resonance imaging) found that areas of the brain associated with the processing of emotions and social thinking showed higher activity while participants were criticised compared to when they were at rest. The authors propose that these brain regions are activated during criticism in order for participants to interpret what is underlying the criticism in order to understand how they can then adapt their behaviour: the participants become more externally orientated.

From this one could hypothesise, that externally orientated clients, as opposed to internally orientated, are more likely to be dedicating brain power to deciphering and responding to their coach’s evaluation rather than exploring their own internal thoughts and desires. Furthermore, a review of physiological studies [94] found that evaluation by others leads to an elevated level of the stress hormone cortisol; again a rather undesired reaction for a coaching client. So non-judgmental attention has been found to not only relax a client but also keep them internally focused.

Another coaching technique, paraphrasing, is a specific aspect of empathy that is used immensely in coaching, mediation and conflict resolution. In one novel neuroimaging study [95] they investigated the impact of paraphrasing on participants discussing a current experience of social conflict. The study found that paraphrasing resulted in participants feeling like they were understood and induced positive emotions. The opposite was true for the un-empathetic intervention in which the interviewer conveyed she did not understand or could not relate to the participant’s situation. The fMRI data showed that different parts of the social cognitive network of the brain were activated for paraphrasing when compared to the un-empathetic response. Although it may seem obvious that showing empathy to coachees and understanding their situations is beneficial during coaching, this study provides an evidence base as to why the use of empathy is so effective.

Coaching is becoming widespread amongst organisations and it is important that the academic research domain understand how coaching is effective as this will enable organisations to maximise and increase the effectiveness of this intervention. Neuroimaging techniques and the field of neuroscience are growing exponentially and although no substantiated research currently exists with coaching and neuroscience that is not to say that this will be the case in the future.

#### Recommendations for Future Research

Based on the findings in this review, we recommend the following best practices for practitioners looking to carry out coaching research. We would also encourage the establishment of an independent working group. The working group would consist of coaches, academics, organisations that use coaching and any stakeholders with an interest in coaching research. The working group would then collaborate and put together a set of guidelines and recommendations—best practices—for conducting sound research that is experimentally rigorous but also provides insight to organisations and practitioners of coaching. These best practices would be freely available to everyone and consist of methodological and statistical procedures, minimum sample sizes, a set of outcome variables both for the individual and the organisation to maintain consistency and to measure objective and distal outcomes. It would provide a source of help and knowledge individuals can leverage to produce sound and applicable coaching research. Furthermore, a goal of this working group would be to create pairings and collaborations between practitioners and academics and different schools of coaching to foster discussion and solutions to the research issues highlighted in this review. Researching the effectiveness of coaching is difficult but it is fundamental to help the industry grow and as a whole will be beneficial to all involved.

#### Best research practices for practitioners

- Pre, post and longitudinal assessment
- Utilise relevant individual, organisational and distal outcomes
- Utilise objective or multisource ratings of outcome variables
- Control group
  - Potentially utilising wait listed participants (see Mackie [46])
- Adequate sample size
  - Assuming a *t*-test analysis between two independent groups (the coached group and the control group) the total sample sizes (assuming an allocation of 1) that result in an adequate power level would be: 52 participants for a large effect (.8), 128 participants for a medium effect (.5) and 352 participants for a small effect. This was calculated using G*Power 3.1 [96].

### Conclusion

Having reviewed quantitative studies over the last decade, encompassing both control group and non-control studies that assessed the efficacy of business, executive and leadership coaching interventions with employed adults in organisational settings, this study has found that a number of individual-level outcome measures have been found to increase through the use of coaching, including well-being, career satisfaction and goal attainment. Organisational level outcomes have been less explored, but initial results point to coaching impacting peer and subordinate ratings of coachees’ leadership behaviours and having positive effects on those that work close to coachees. The evidence pertaining to variables that potentially moderate or mediate the effectiveness of coaching interventions within this population is severely limited but does implicate the existence of moderators and mediators that need to be explored with further research.

A number of issues related to research design and methodology have been highlighted in this review and best practices for researchers have been discussed. It is important that researchers continue and strengthen their research efforts into the efficacy of coaching. It is unjustifiable that an intervention so extensively used throughout organisations does not have a foundation of academically rigorous effectiveness research. This research will not only help practitioners of coaching improve and maximise the effectiveness of their interventions but also provide organisations with the much needed information about how and when to employ coaching so it is beneficial to both the individual and the organisation as a whole.

## Supporting Information

S1 Table PRISMA checklist of items to include when reporting a systematic review or meta-analysis. (PDF)
